# Tumor microenvironment-related multigene prognostic prediction model for breast cancer

**DOI:** 10.18632/aging.203845

**Published:** 2022-01-20

**Authors:** Kai Hong, Yingjue Zhang, Lingli Yao, Jiabo Zhang, Xianneng Sheng, Yu Guo

**Affiliations:** 1 Medicine School, Ningbo University, Jiangbei, Ningbo 315211, Zhejiang, China; 2 Department of Molecular Pathology, Division of Health Sciences, Graduate School of Medicine, Osaka University, Suita, Osaka 565–0871, Japan; 3 Department of Thyroid and Breast Surgery, Ningbo City First Hospital, Haishu, Ningbo 315010, Zhejiang, China

**Keywords:** breast cancer, biomarkers, differentially expressed genes, risk score, tumor microenvironment

## Abstract

Background: Breast cancer is an invasive disease with complex molecular mechanisms. Prognosis-related biomarkers are still urgently needed to predict outcomes of breast cancer patients.

Methods: Original data were download from The Cancer Genome Atlas (TCGA) and the Gene Expression Omnibus (GEO). The analyses were performed using perl-5.32 and R-x64-4.1.1.

Results: In this study, 1086 differentially expressed genes (DEGs) were identified in the TCGA cohort; 523 shared DEGs were identified in the TCGA and GSE10886 cohorts. Eight subtypes were estimated using non-negative matrix factorization clustering with significant differences seen in overall survival (OS) and progression-free survival (PFS) (*P* < 0.01). Univariate Cox analysis and least absolute shrinkage and selection operator (LASSO) regression analysis were performed to develop a related risk score related to the 17 DEGs; this score separated breast cancer into low- and high-risk groups with significant differences in survival (*P* < 0.01) and showed powerful effectiveness (TCGA all group: 1-year area under the curve [AUC] = 0.729, 3-year AUC = 0.778, 5-year AUC = 0.781). A nomogram prediction model was constructed using non-negative matrix factorization clustering, the risk score, and clinical characteristics. Our model was confirmed to be related with tumor microenvironment. Furthermore, DEGs in high-risk breast cancer were enriched in histidine metabolism (normalized enrichment score [NES] = 1.49, *P* < 0.05), protein export (NES = 1.58, P < 0.05), and steroid hormone biosynthesis signaling pathways (NES = 1.56, P < 0.05).

Conclusions: We established a comprehensive model that can predict prognosis and guide treatment.

## INTRODUCTION

Breast cancer is the most commonly diagnosed malignancy and cause of cancer-related deaths in females [1]. According to the statistics from 2020, approximately 276,480 female breast cancers were diagnosed in the US, and 42,170 patients are expected to die from breast cancer [2]. Despite early diagnosis, abundant treatments, and a decline in the mortality rate of this disease over the past year, patients with advanced and metastatic breast cancer still experience a high mortality rate [3]. Therefore, an effective risk model for breast cancer can play a vital role in individualized therapy.

Breast cancer is strongly correlated with changes in gene status, such as amplifications, downregulation, and mutations. Traditionally, according to immunohistochemistry for estrogen receptor (ER), progesterone receptor (PR), and human epidermal growth factor receptor2 (HER2), breast cancer is classified into the luminal A, luminal B, HER2-positive, and triple-negative subtypes [4]. However, with further exploration of the molecular mechanisms of breast cancer, more detailed molecular types have been presented, such as luminal A, luminal B, HER2-enriched, basal-like, normal-like, and claudin-low [5]. Different subtypes have been confirmed to have different prognoses and drug responses. With advances in statistical analysis, multigene signatures are widely used to predict patient prognosis and drug response [6, 7]. Some multigene prediction models, such as the Oncotype DX 21-gene test, Prediction Analysis of Microarray 50, and 70-gene signature (MammaPrint), have been applied in the clinic [8–10]. The Oncotype DX 21-gene test can evaluate the tumor recurrence and predict chemotherapy responses in patients with ER-positive breast cancer. MammaPrint signature and PAM50 can improve prognostic prediction in breast cancer patients. Nonetheless, despite their high power, these tools only consider gene status, and thus, a model with comprehensive consideration of additional factors is urgently needed.

Breast cancer outcomes are significantly related to factors, such as tumor size, tumor stage, lymph node status, age, tumor tissue receptor status, and gene status. To effectively evaluate prognoses of breast cancer and predict drug responses to guide treatment, we constructed a comprehensive prediction model with varied factors. In this study, we identified differentially expressed genes (DEGs) from The Cancer Genome Atlas (TCGA) and Gene Expression Omnibus (GEO) and performed non-negative matrix factorization (NMF) clustering and a least absolute shrinkage and selection operator (LASSO) regression analysis to construct a nomogram prediction model. We also explored the correlations and potential signaling pathways and discuss a possibly internal mechanism of breast cancer.

## MATERIALS AND METHODS

### Data acquisition

Gene expression, tumor mutation burden, and clinical information datasets of breast cancer were obtained from The Cancer Genome Atlas (TCGA, https://portal.gdc.cancer.gov/) and Gene Expression Omnibus (GEO, https://www.ncbi.nlm.nih.gov/geo/) databases in October 2021. We selected 1208 samples, including 1096 breast cancer samples and 112 normal samples from The Cancer Genome Atlas Breast Invasive Carcinoma (TCGA-BRCA) program. After searching the datasets with more than 150 human breast cancer samples with complete expression profile data, we selected the GSE10886 dataset from the GEO [9].

### Analysis of differentially expressed genes

**Figure 1:**
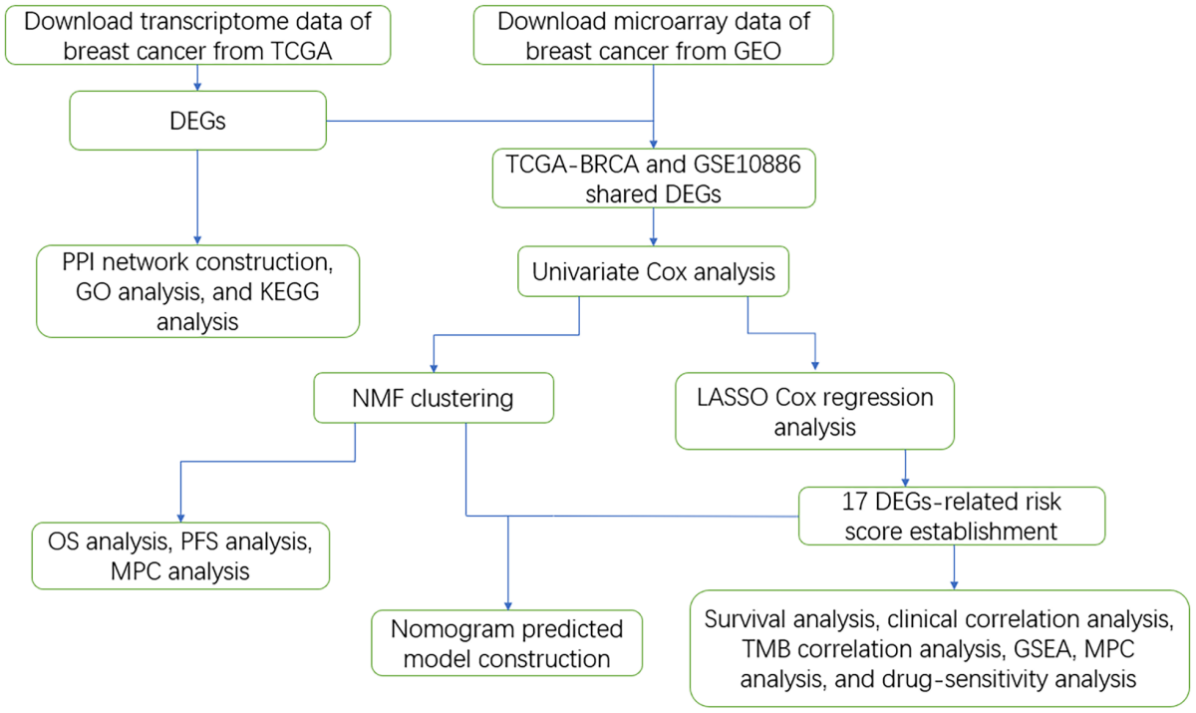
Work flowchart of the study.

To identify the DEGs, we used a multi-step approach (Figure 1). The limma and sva R packages were used for the differential expression analysis (log fold change [FC] >1, false discovery rate [FDR] < 0.05) and batch correction for the TCGA-BRCA [11–15] (Supplementary File 1).

### Protein-protein interaction network and enrichment analysis

The protein-protein interaction (PPI) network for DEGs was constructed using the STRING website tool (https://string-db.org/); the high confidence genes were conserved (interaction score ≥ 0.7), and hub nodes were visualized by R-x64-4.1.1 (Supplementary File 1). Gene ontology (GO) and Kyoto Encyclopedia of Genes and Genomes (KEGG) enrichment analyses were conducted using the clusterprofiler R package, where P < 0.05 [16–18] (Supplementary File 1).

### Non-negative matrix factorization clustering

First, we intersected the TCGA-BRCA and GSE10886 data to obtain the shared DEGs; then, we analyzed the DEGs using the survival and NMF R packages (Supplementary File 1), through which eight distinct subtypes were identified according to the cophenetic correlation coefficient for the cluster number from 2–10 [19]. Second, the overall survival (OS) and progression-free survival (PFS) differences among these subtypes, as observed by Kaplan-Meier (K-M) analysis and log-rank test, were analyzed using the survival and survminer R packages [20] (Supplementary File 1). Third, the analysis of microenvironment cell populations (MCP) differences among these subtypes was performed using the limma, ggpubr, and MCPcounter R packages [21] (Supplementary File 1).

### Nomogram model establishment

Based on the DEG data, a univariate Cox analysis was performed to identify the survival related genes (*P* < 0.05). A modified LASSO regression analysis was conducted to find the genes most relevant to the OS of breast cancer patients (*P* < 0.05) [22–24] (Supplementary File 1). To verify the accuracy of our risk score predictor, analyses of training, and test groups were performed using data that were randomly obtained from the whole group. The predictive ability of the risk score was evaluated by survival probability curve, receiver operating characteristic (ROC) curve, and the area under the curve (AUC) [25] (Supplementary File 1). The risk computing formula is as follows: Risk score $=\sum_{i=1}^{n}Coef\;(i)*Expr\;(i)$. In addition, univariate and multivariate analyses were performed to demonstrate the independent predictive ability of the risk score. A nomogram prediction model was established using clinical characteristics, NMF clustering, and risk score to predict the survival of breast cancer patients; the nomogram-predicted probability of the 1-, 3-, and 5-year OS is shown by the calibration curve [26]. To identify the superiority of the nomogram, a decision curve analysis (DCA) was conducted [27] (Supplementary File 1). The R packages survival, survminer, caret, glmnet, timeROC, ggDCA, regplot, and rms were used for these analyses (Supplementary File 1).

### Gene set enrichment analysis

Gene set enrichment analysis (GSEA) was conducted between low- and high-risk score subsets [28] (Supplementary File 1). The “c2.cp.kegg.v7.4.symbols.gmt” was obtained from (https://www.gsea-msigdb.org/gsea/index.jsp). Signaling pathways with *P* < 0.05 and FDR < 0.05 were considered enriched.

### Clinical characteristics, genes, immune cells, and tumor mutation burden correlation analysis

The correlations between risk score and clinical characteristics, known breast cancer genes, immune cells, and tumor mutation burden (TMB) are shown through box plots, correlation matrix, and circular plot generated by ggpubr, corrplot, and circlize R packages, respectively [29] (Supplementary File 1).

### Relevance analysis between NMF clustering and risk score

To further explore the relevance between our novel typing mode and independent predictive factors, a Sankey diagram was plotted using ggalluvial, ggplot2, and dplyr R packages [30–32] (Supplementary File 1).

### Immunohistochemistry

Immunohistochemistry results for risk score related DEGs in breast cancer were obtained from The Human Protein Atlasdatabase (THPA, https://www.proteinatlas.org/).

### Chemotherapeutic and immunotherapeutic response prediction

We predicted the drug response in breast cancer patients based on the Genomics of Drug Sensitivity in Cancer database [33]. Six common chemotherapeutic and immunotherapeutic drugs (paclitaxel, cytarabine, camptothecin, lapatinib, erlotinib, and gefitinib) were selected for the analysis. The R package pRRophetic was used to conduct the analysis [34] (Supplementary File 1). The inhibitory concentration (IC_50_) was assessed to determine the drug sensitivities. ComBat was used to adjust for batch effects, and the average expression was calculated for repeated genes.

### Statistical analysis

Statistical analysis was performed using R-x64-4.1.1 and perl-5.32 (Supplementary File 1). Data are presented as means ± standard deviation. Differences with *P* < 0.05 and FDR < 0.05 were considered statistically significant.

### Data availability statement

All data are available from online database included TCGA (https://portal.gdc.cancer.gov/), GEO (https://www.ncbi.nlm.nih.gov/geo/), THPA (https://www.proteinatlas.org/), and GSEA (https://www.gsea-msigdb.org/gsea/index.jsp).

### Ethical statement

TCGA, GEO, THPA, and GSEA belong to public databases. Patients involved in them all had ethical approval. All analyses of us were based on them, therefore, no ethical issues existed.

## RESULTS

### Differentially expressed genes in breast cancer

**Figure 2:**
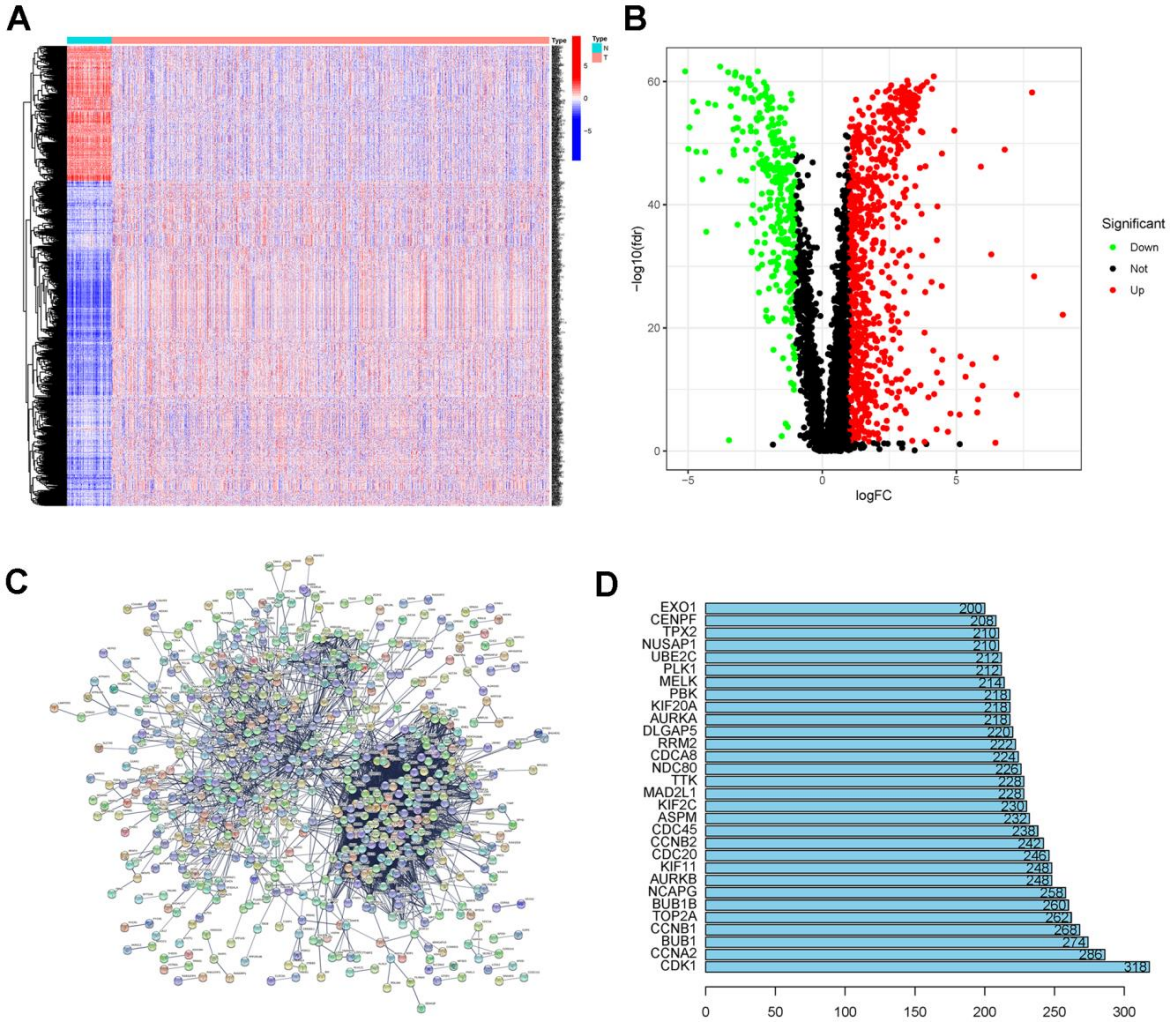
(**A**, **B**) DEG analysis for TCGA-BRCA. (**C**) PPI network of the TCGA-BRCA DEGs. (**D**) Hub node numbers in the PPI network of the TCGA-BRCA DEGs. Abbreviations: DEGs: differentially expressed genes; TCGA-BRCA: The Cancer Genome Atlas Breast Invasive Carcinoma; PPI network: protein-protein interaction network.

The expression of 1086 DEGs was founded in the TCGA-BRCA cohort by comparing breast cancer tissue (1096 tumor samples) with normal tissue (112 normal breast samples) (Figure 2A, 2B).

### Protein-protein interaction network analysis and enrichment analysis

**Figure 3:**
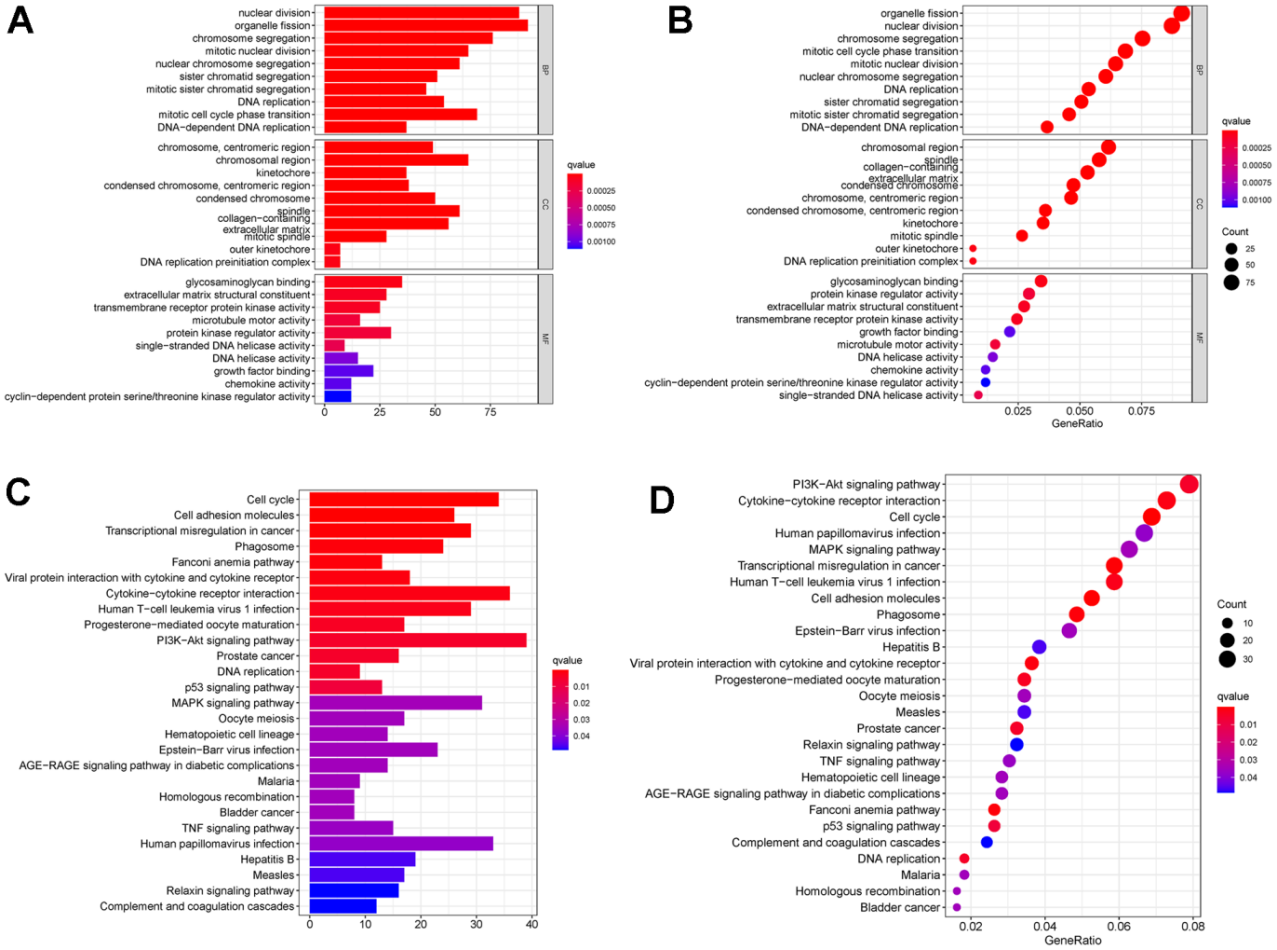
(**A**, **B**) GO analysis for TCGA-BRCA DEGs. (**C**, **D**) KEGG analysis for TCGA-BRCA DEGs. Abbreviations: GO: Gene Ontology; KEGG: Kyoto Encyclopedia of Genes and Genomes; DEGs: differentially expressed genes; TCGA-BRCA: The Cancer Genome Atlas Breast Invasive Carcinoma.

A PPI network was constructed for the TCGA-BRCA DEGs (Figure 2C). The top thirty genes ranked by connectivity degree are shown in Figure 2D. The results showed that CDK1 was the most significant gene with a connectivity degree of 318, followed by CCNA2 (degree = 286), BUB1 (degree = 274), CCNB1 (degree = 268), and TOP2A (degree = 262). Biological process analysis showed that “nuclear division,” “organelle fission,” “chromosome segregation,” “mitotic cell cycle phase transition,” and “mitotic nuclear division” were significantly relevant to the DEGs (Figure 3A, 3B). Cellular component analysis demonstrated the significantly association between the DEGs with “chromosomal region,” “spindle,” “collagen-containing extracellular matrix,” “condensed chromosome,” and “chromosome, centromeric region” (Figure 3A, 3B). According to the molecular function analysis, the DEGs were enriched in “glycosaminoglycan binding,” “protein kinase regulator activity,” “extracellular matrix structural constituent,” “transmembrane receptor protein kinase activity,” and “growth factor binding” (Figure 3A, 3B). In the KEGG analysis, the top five enriched pathways were “PI3K-Akt signaling pathway,” “cytokine-cytokine receptor interaction,” “cell cycle,” “human papillomavirus infection,” and “MAPK signaling pathway” (Figure 3C, 3D).

### Non-negative matrix factorization clustering analysis

**Figure 4:**
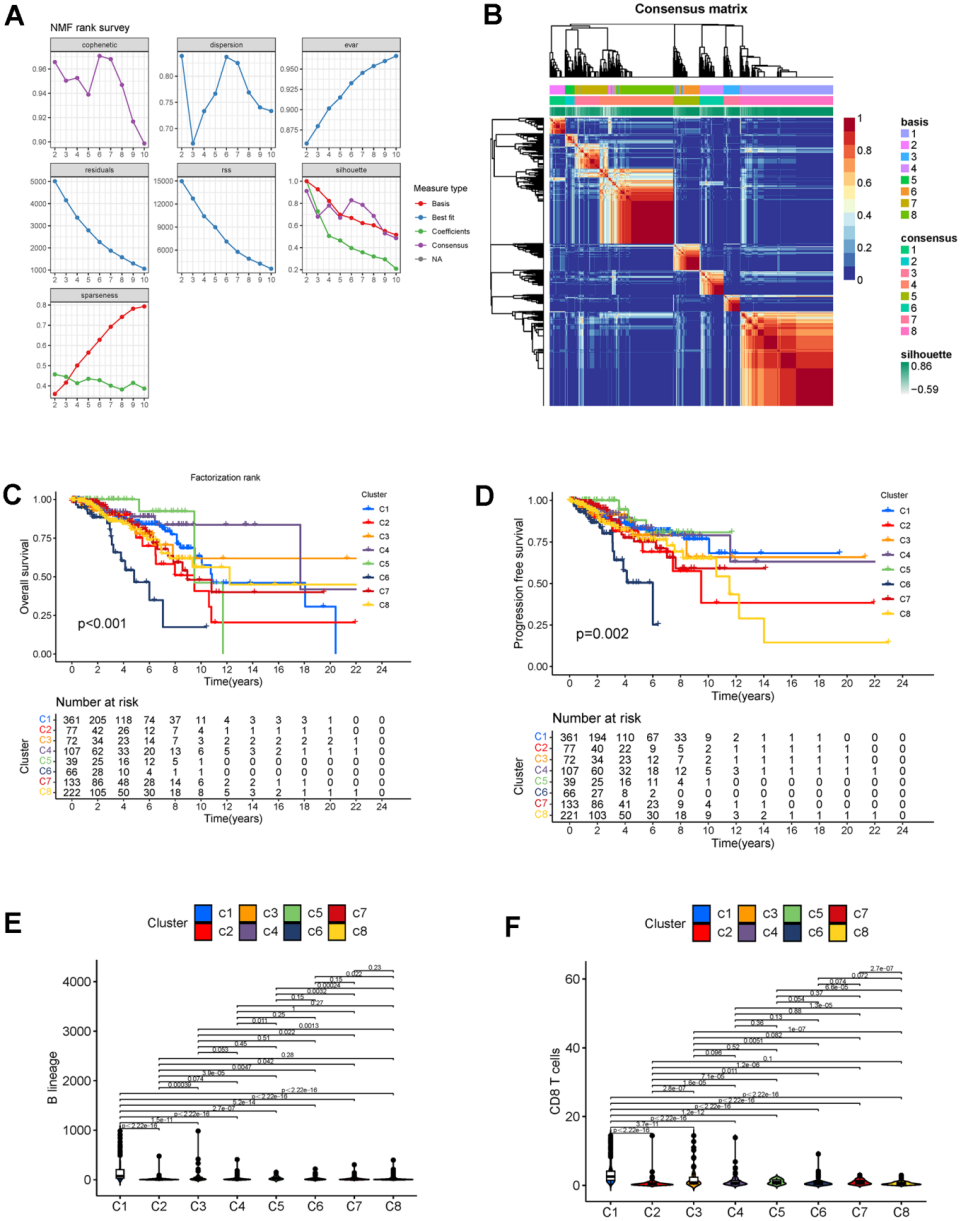
(**A**) Factorization rank for 2–10 clusters. (**B**) Heatmap of the gene expression of eight clusters. (**C**, **D**) K-M curves for OS and PFS in different subtypes. (**E**) B lineage cell infiltration in different subtypes. (**F**) CD8+ T cell infiltration in different subtypes. Abbreviations: K-M: Kaplan-Meier; OS: overall survival; PFS: progression-free survival.

**Figure 5:**
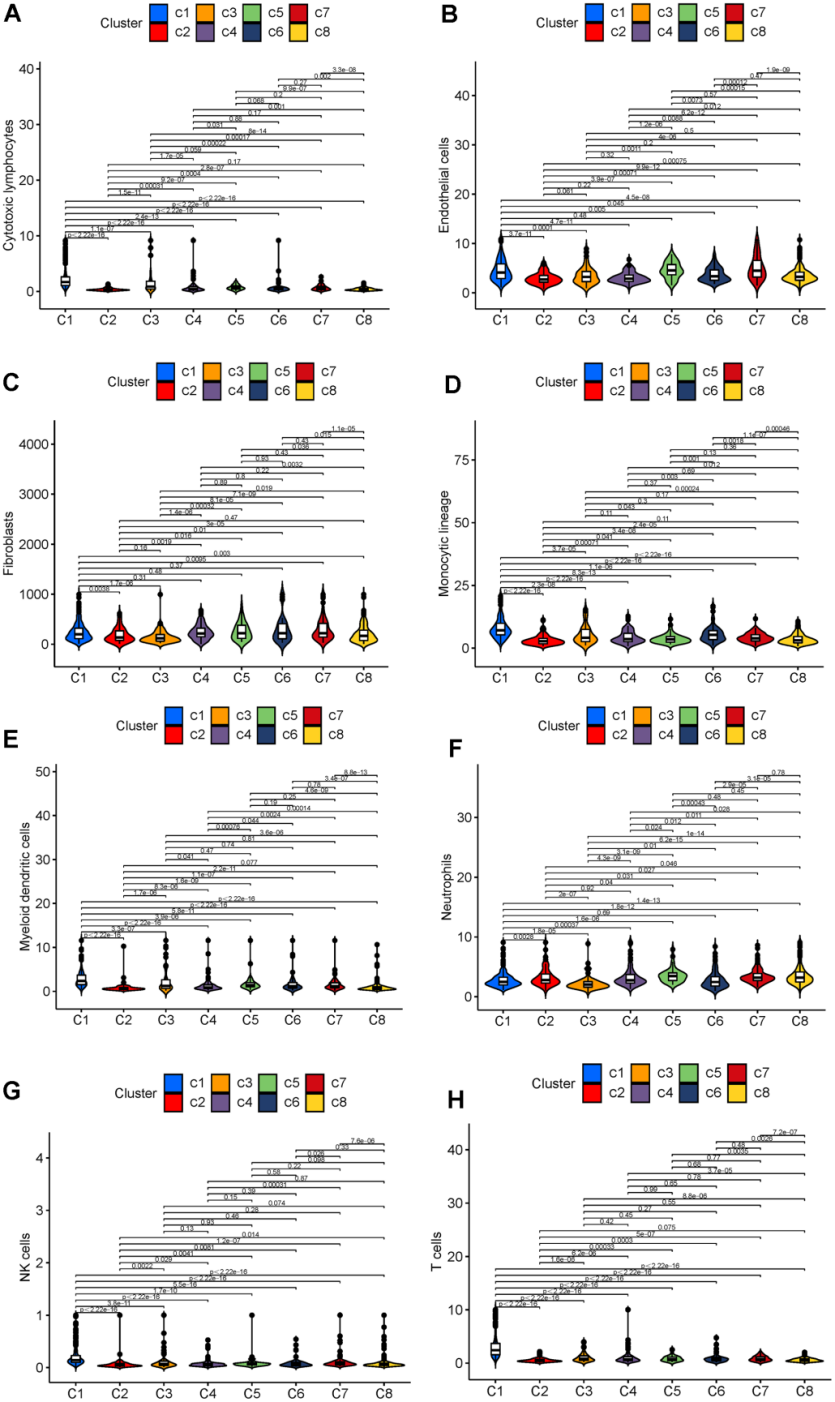
(**A**) Cytotoxic lymphocyte infiltration in different subtypes. (**B**) Endothelial cell infiltration in different subtypes. (**C**) Fibroblast infiltration in different subtypes. (**D**) Monocytic lineage cell infiltration in different subtypes. (**E**) Myeloid dendritic cell infiltration in different subtypes. (**F**) Neutrophil infiltration in different subtypes. (**G**) NK cell infiltration in different subtypes. (**H**) T cell infiltration in different subtypes.

First, a univariate Cox regression analysis was performed using the data of the DEGs to increase the robustness of our cluster (*P* < 0.01). The NMF algorithm was then used to cluster the breast cancer cases based on gene expression, survival time, and survival status. We identified the optimal k value of 8 with the cophenetic correlation coefficients, and confirmed that the optimal cluster number was 8 (Figure 4A). As shown in Figure 4B, the boundaries of the eight subtypes (C1-C8) are clear, which indicates that the clustering is relatively reliable. According to the OS and PFS curves (Figure 4C, 4D), C4 and C5 breast cancer is associated with the best prognosis, and C6 breast cancer patients have the worst (*P* < 0.01). MCP counting for eight tissue-infiltrating immune cell types (B lineage, monocytic lineage, cytotoxic lymphocytes, neutrophils, myeloid dendritic cells, NK cells, T cells, and CD8+ T cells) and two stromal cell types (fibroblasts and endothelial cells) was performing using the R package MCPcounter (Supplementary File 1). We found that C1 had the greatest abundance of these cells, except neutrophils, in the microenvironment (Figures 4, 5). Moreover, fibroblasts, endothelial cells, and neutrophils were significantly high in C5, while tissue-infiltrating immune cells were significantly low in C6 (Figure 5B, 5C, 5F).

### Construction of the prognostic model

**Figure 6:**
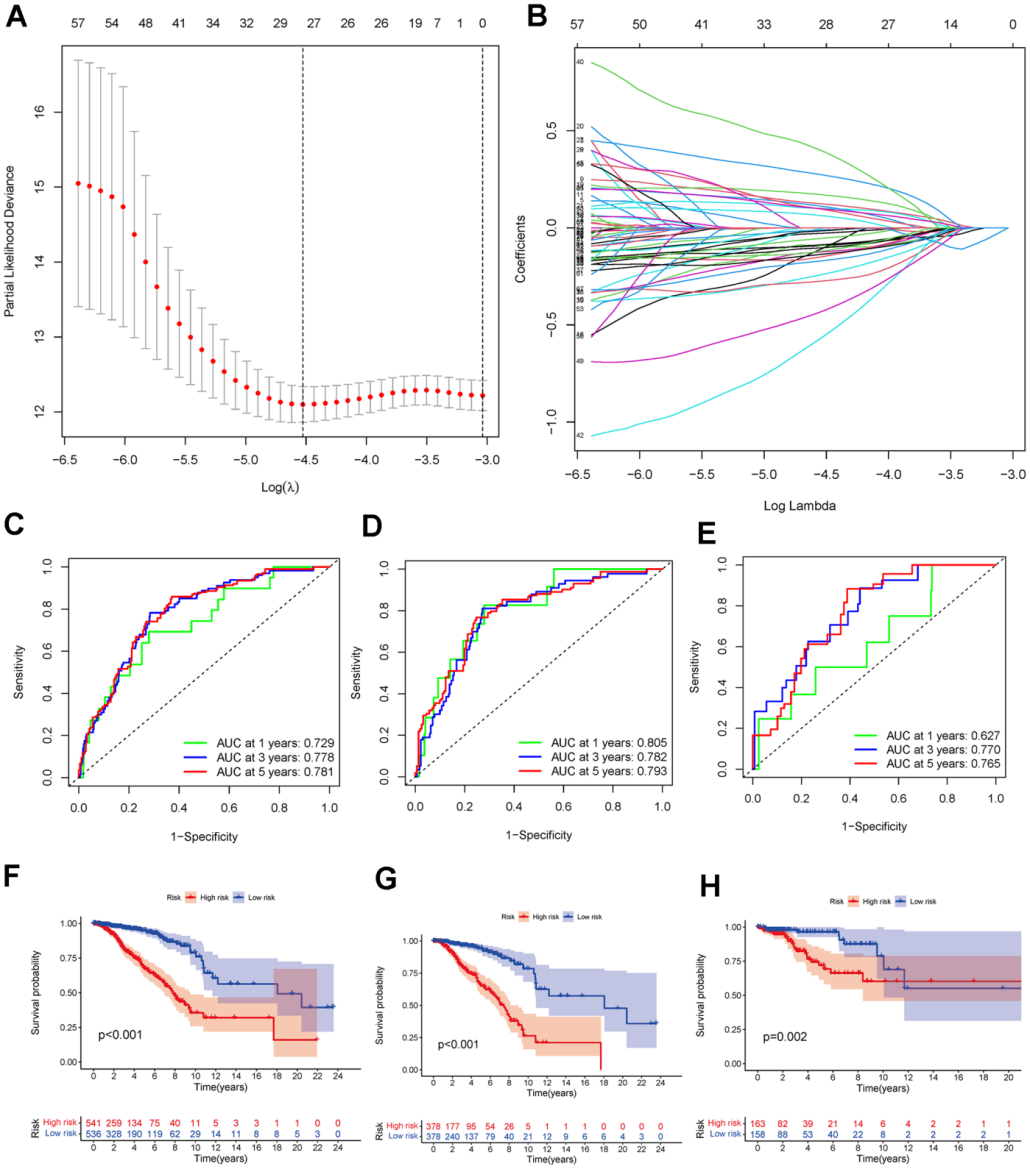
(**A**, **B**) The LASSO regression analysis identified 17 DEGs mostly related to prognosis. (**C**–**E**) The 1-, 3-, and 5-year ROC analyses in the whole TCGA-BRCA cohort, TCGA-BRCA training cohort, and TCGA-BRCA test cohort. (**F**–**H**) K-M curves of OS for low- and high-risk breast cancers in the whole TCGA-BRCA cohort, the TCGA-BRCA training cohort, and TCGA-BRCA test cohort. Abbreviations: LASSO: least absolute shrinkage and selection operator; DEGs: differentially expressed genes; K-M: Kaplan-Meier; TCGA-BRCA: The Cancer Genome Atlas Breast Invasive Carcinoma; ROC: receiver operating characteristic.

**Figure 7:**
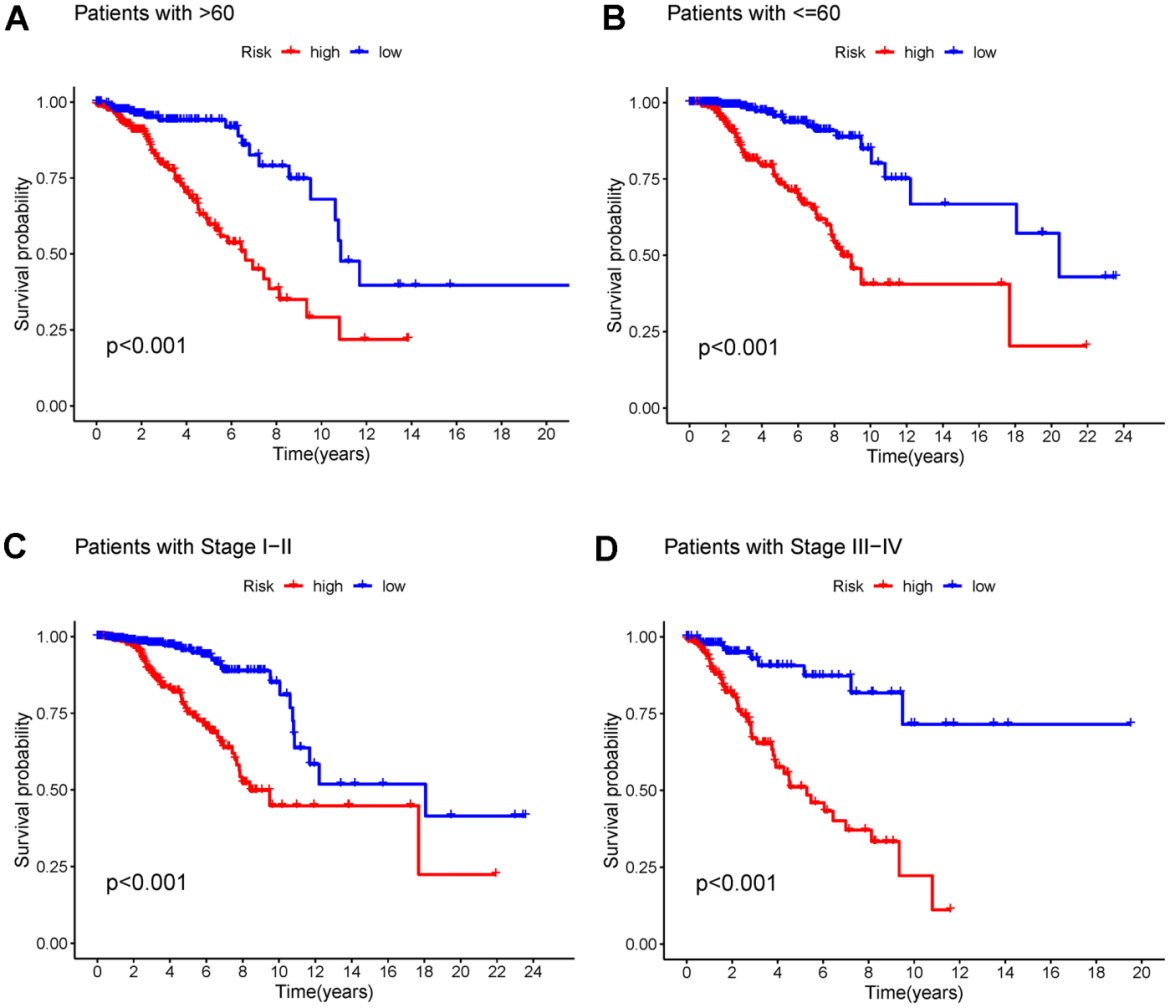
(**A**, **B**) K-M curves of OS for breast cancer patients age >60 and age ≤60 in the low- and high-risk groups. (**C**, **D**) K-M curves of OS for stage I-II and stage III-IV breast cancer in the low- and high-risk groups. Abbreviations: K-M: Kaplan-Meier; OS: overall survival.

**Figure 8:**
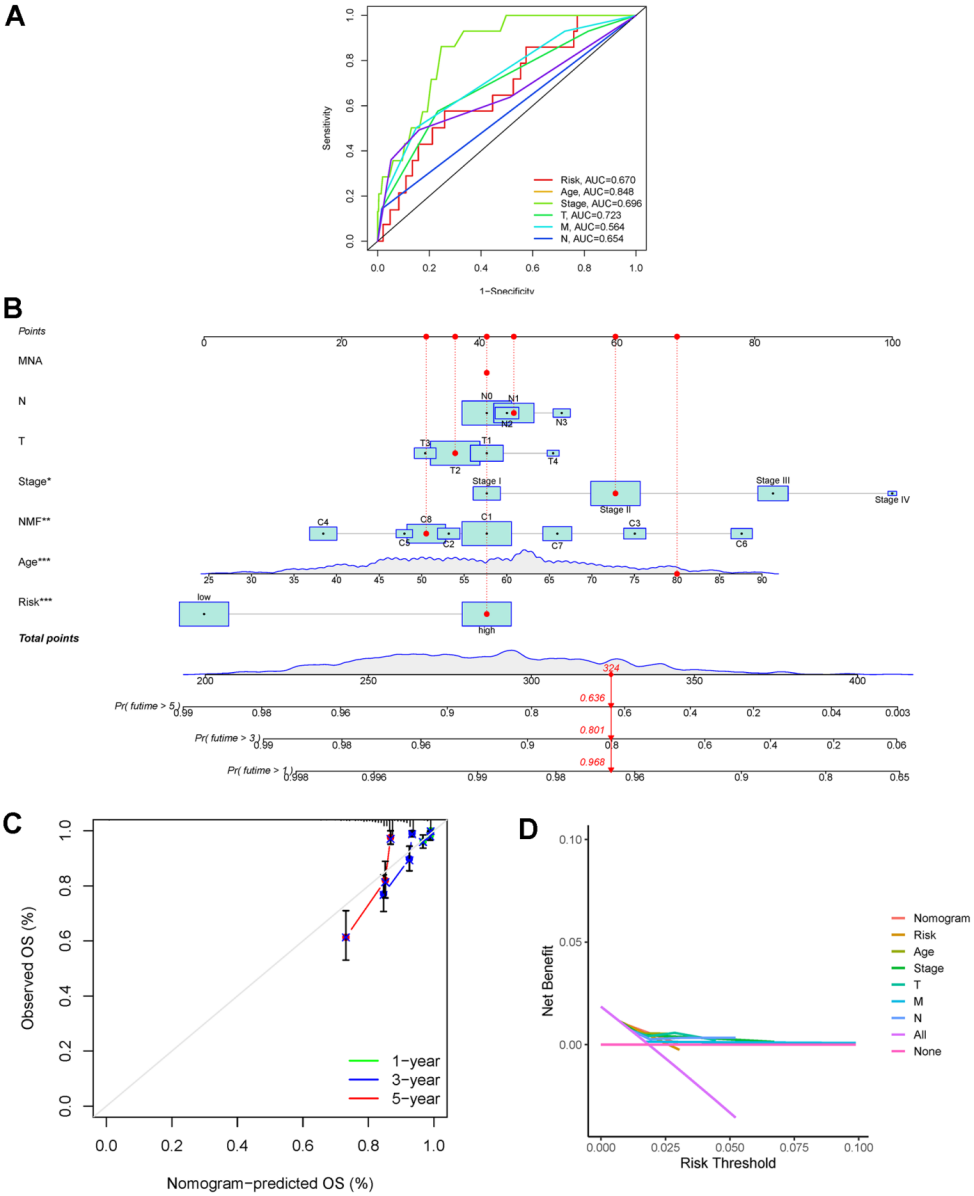
(**A**) Calculation of the AUC for risk score, age, stage, T, M, and N. (**B**) Nomogram-predicted model for breast cancer. (**C**) Calibration plots for 1-, 3-, and 5-year survival probabilities. (**D**) DCA of the nomogram, risk score, age, stage, T stage, M stage, and N stage. Abbreviations: AUC: area under the curve; DCA: decision curve analysis.

Based on the 523 DEGs, a univariate Cox analysis was first performed to increase the stability of the results. Subsequently, LASSO regression analysis was performed to identify the 17 DEGs included in the risk assessment model (Figure 6A, 6B). The risk score was calculated according to the following formula: (0.186995130166584) * ExprWNT7B + (−0.367068269289688) * ExprBCL2A1 + (0.361699290337736) * ExprULBP2 + (−0.26235003273008) * ExprLEF1 + (0.386283757445905) * ExprGABRQ + (−0.140710133664929) * ExprFXYD3 + (0.183333746976684) * ExprSCG2 + (−0.388494904628374) * ExprFOXJ1 + (−0.22258520830588) * ExprTP63 + (−0.450564126981243) * ExprRYR1 + (0.646038331120338) * ExprFEZ1 + (−1.23444095440368) * ExprNRG1 + (0.186015162843612) * ExprRGS4 + (−0.711499870665385) * ExprNFE2 + (0.174340252592255) * ExprHOXC13 + (−0.249218472277074) * ExprMMP25 + (−0.331805787206587) * ExprDTX1. The cut-off value used to divide patients into the low- and high-risk breast cancer was −2.025. To further verify the accuracy of the risk score predictor, ROC analysis was performed (Figure 6C–6E). The 1-, 2-, and 3-year AUC values for the whole TCGA cohort were 0.729, 0.778, and 0.781, respectively, while the 1-, 2-, and 3-year AUC values for the TCGA training cohort were 0.805, 0.782, and 0.793, respectively, and the 1-, 2-, and 3-year AUC values for the TCGA test cohort were 0.627, 0.770, and 0.765, respectively. According to the K-M plotter, survival differences between the low- and high-risk breast cancer groups in the whole TCGA cohort, TCGA training cohort, and TCGA test cohort were significant (*P*<0.01) (Figure 6F–6H). In addition, significant differences were also observed in the age >60, age ≤60, stage I-II, and stage III-IV subsets (Figure 7). Moreover, the univariate and multivariate Cox analyses of the relationship between the risk score and clinical characteristics demonstrated that the risk score was an independent predictor of breast cancer (univariate Cox regression: hazard ratio [HR] = 1.14, 95% confidence interval [CI] = 1.10 − 1.17, *P* < 0.01; multivariate Cox regression: hazard ratio = 1.13, 95% confidence interval = 1.09 − 1.17, *P* < 0.01) (Table 1). Although the risk score is an independent factor, compared with other clinical characteristics, the AUC value of the risk score was not the highest (Figure 8A). To further improve predictive ability, a nomogram model was constructed using the clinical characteristics, NMF clustering, and risk score. According to the nomogram model, each patient has a total score that can predict the 1-, 3-, and 5-year survival rates (Figure 8B). The calibration curves showed that the nomogram-predicted 1-, 3-, and 5-year OS probabilities were close to the actual OS (Figure 8C). Moreover, the results of DCA showed that our nomogram was the best predictor (Figure 8D).

**Table 1:** Univariate and multivariate Cox analyses.

**Univariate Cox analysis**				
**id**	**HR**	**HR.95L**	**HR.95H**	**p-value**
Age	1.034397	1.01977	1.049234	3.25E−06
Stage	2.109207	1.668588	2.666178	4.32E−10
T	1.570708	1.265938	1.94885	4.09E−05
M	6.027352	3.314181	10.96167	3.94E−09
N	1.67166	1.393874	2.004807	3.00E−08
Risk score	1.136899	1.103803	1.170987	1.71E−17
**Multivariate Cox analysis**				
**id**	**HR**	**HR.95L**	**HR.95H**	**p-value**
Age	1.031857	1.016637	1.047306	3.53E−05
Stage	1.450661	0.855984	2.458478	0.166909
T	1.068995	0.787655	1.450826	0.668532
M	1.383885	0.596304	3.211683	0.449431
N	1.305108	0.977835	1.741916	0.070638
Risk score	1.12811	1.091573	1.16587	7.18E−13

HR, hazard ratio; HR.95L, hazard ratio 95% CI low; HR.95H, hazard ratio 95% CI high.

### Potential signaling pathways in the low- and high-risk groups

**Figure 9:**
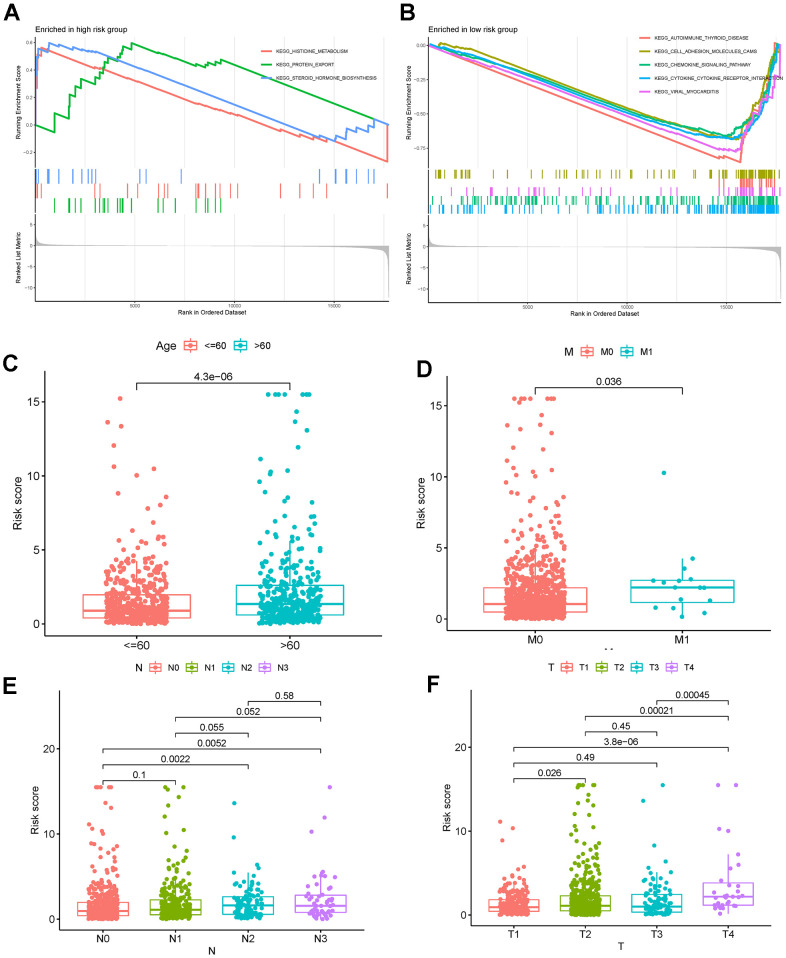
(**A**, **B**) GSEA in low- and high-risk breast cancer. (**C**–**F**) Correlation analyses of the risk score with age, M stage, N stage, and T stage. Abbreviations: GSEA: gene set enrichment analysis.

To further explore the potential signaling pathways of DEGs in the low- and high-risk groups, GSEA was performed. The GSEA results showed that the histidine metabolism signaling pathway (normalized enrichment score [NES] = 1.49, *P* < 0.05), protein export signaling pathway (NES = 1.58, P < 0.05), and steroid hormone biosynthesis signaling pathway (NES = 1.56, P < 0.05) were significantly enriched in the high-risk group (Figure 9A). In contrast, the autoimmune thyroid disease signaling pathway (NES = −1.88, P < 0.05), cell adhesion molecules cams signaling pathway (NES = −1.71, P < 0.05), chemokine signaling pathway (NES = −1.68, P < 0.05), cytokine-cytokine receptor interaction signaling pathway (NES = −1.75, P < 0.05), and viral myocarditis signaling pathway (NES = −1.83, P < 0.05) were enriched in the low-risk group (Figure 9B).

### Factors correlated with the risk score

**Figure 10:**
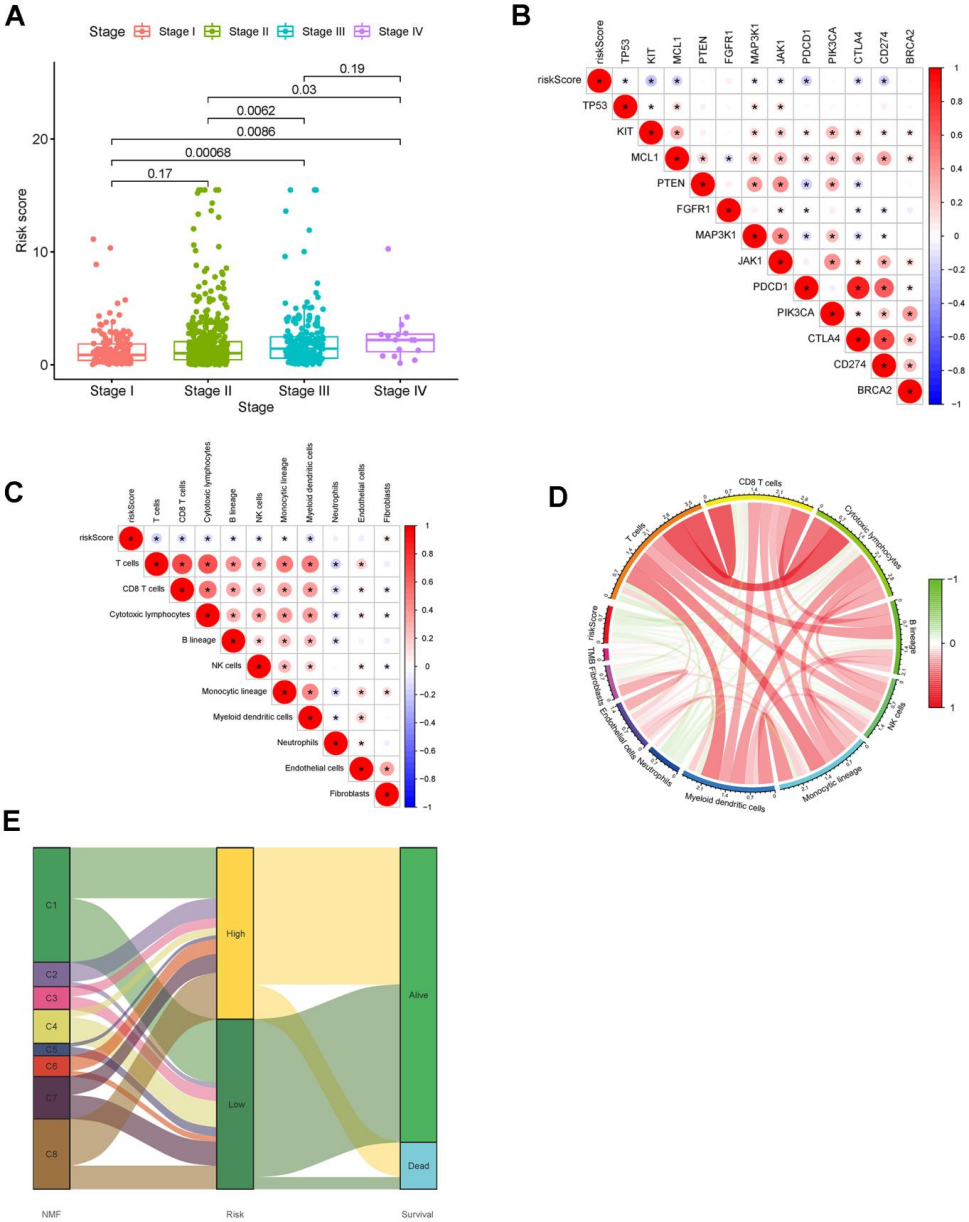
Correlation analysis of (**A**) Risk score and tumor stage. (**B**) Risk score and breast cancer-associated genes. (**C**) Risk score and immune cell infiltration. (**D**) TMB. (**E**) NMF clustering and risk score. Abbreviations: TMB: tumor mutation burden; NMF: non-negative matrix factorization.

We researched the correlations between the risk score and clinical characteristics and found that age (Figure 9C), M stage (Figure 9D), N stage (Figure 9E), T stage (Figure 9F), and clinical stage (Figure 10A) were significantly associated with the risk score. We then selected 12 known breast cancer-related genes and analyzed their correlations with the risk score. As shown in Figure 10B, significant negative correlations existed between the risk score and TP53, KIT, MCL1, MAP3K1, JAK1, PDCD1, CTLA4, and CD274. Infiltrating T cells, CD8 + T cells, cytotoxic lymphocytes, B lineage cells, NK cells, monocytic lineage cells, and myeloid dendritic cells were significantly negatively correlated with the risk score, while the fibroblasts were significantly positive correlated to the risk score (Figure 10C). In addition, we did not find a significant correlation between the risk score and TMB (Figure 10D).

### Correlation between NMF clustering and the risk score

To connect the NMF clustering and risk score, we generated a Sankey diagram (Figure 10E). The plot showed that more dead patients had high-risk scores, which further demonstrated the reliability of the risk score. Furthermore, the C2 and C6 subtypes mainly contained high-risk scores breast cancer and had the worse prognoses according to the survival curves, while the C4 and C5 subtypes included more low-risk breast cancer had better prognoses. These results demonstrated the accuracy of both the novel typing mode and the predictive factor.

### Validation of the risk score-relevant genes in breast cancer tissue

**Figure 11:**
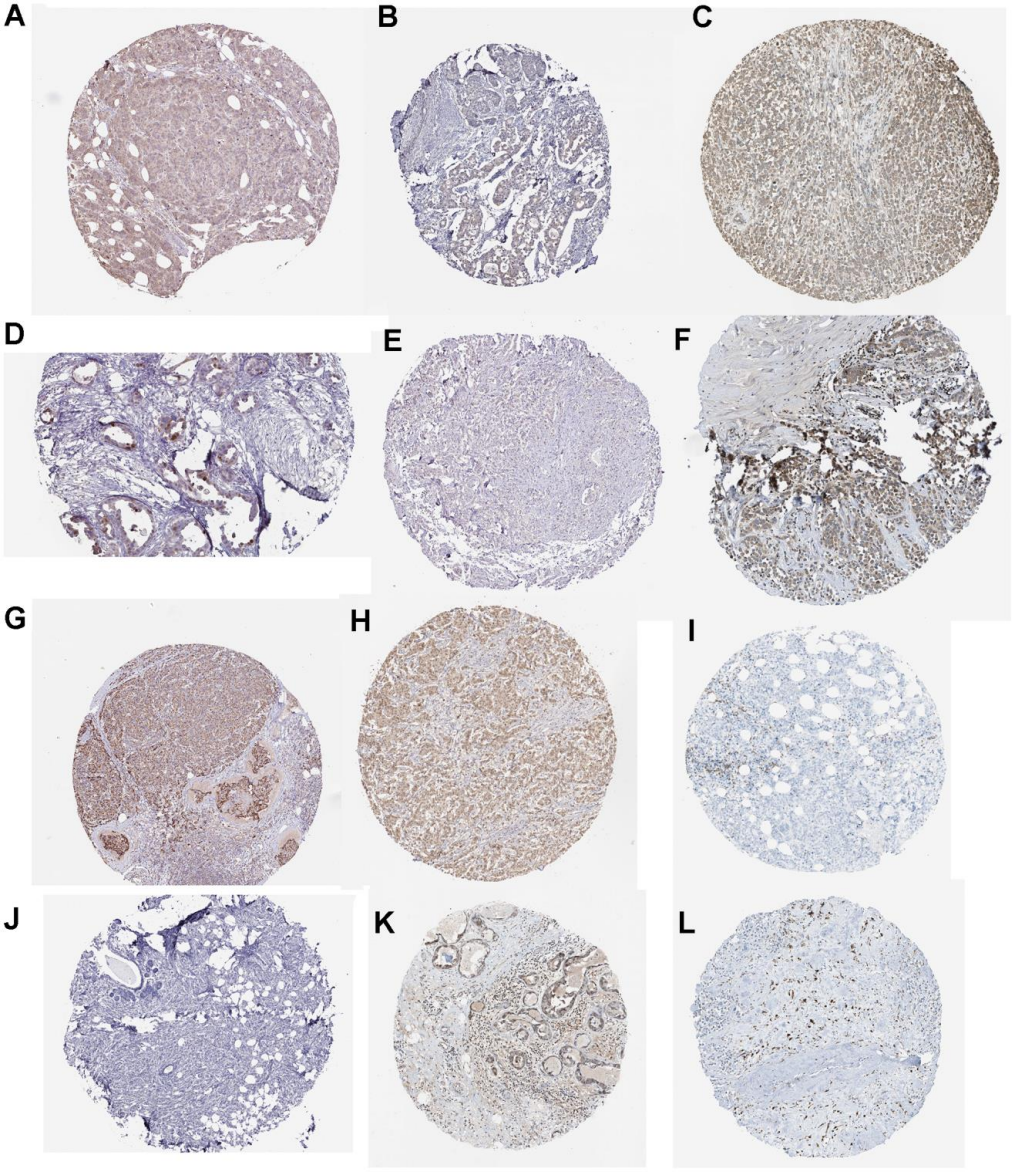
Immunohistochemistry for (**A**) DTX1 (https://www.proteinatlas.org/ENSG00000135144-DTX1/pathology/breast+cancer#imid_18106719). (**B**) FEZ1 (https://www.proteinatlas.org/ENSG00000149557-FEZ1/pathology/breast+cancer#imid_20550866). (**C**) FXYD3 (https://www.proteinatlas.org/ENSG00000089356-FXYD3/pathology/breast+cancer#imid_3109719). (**D**) FOXJ1 (https://www.proteinatlas.org/ENSG00000129654-FOXJ1/pathology/breast+cancer#imid_18961115). (**E**) HOXC13 (https://www.proteinatlas.org/ENSG00000123364-HOXC13/pathology/breast+cancer#imid_15073368). (**F**) LEF1 (https://www.proteinatlas.org/ENSG00000138795-LEF1/pathology/breast+cancer#imid_913343). (**G**) MMP25 (https://www.proteinatlas.org/ENSG00000008516-MMP25/pathology/breast+cancer#imid_9651206). (**H**) NFE2 (https://www.proteinatlas.org/ENSG00000123405-NFE2/pathology/breast+cancer#imid_927392). (**I**) NRG1 (https://www.proteinatlas.org/ENSG00000157168-NRG1/pathology/breast+cancer#imid_3118350). (**J**) SCG2 (https://www.proteinatlas.org/ENSG00000171951-SCG2/pathology/breast+cancer#imid_20497272). (**K**) TP63 (https://www.proteinatlas.org/ENSG00000073282-TP63/pathology/breast+cancer#imid_143251). (**L**) ULBP2 (https://www.proteinatlas.org/ENSG00000131015-ULBP2/pathology/breast+cancer#imid_5232353).

Immunohistochemistry demonstrated the expression of DTX1, FEZ1, FOXJ1, FXYD3, HOXC13, LEF1, MMP25, NFE2, NRG1, SCG2, TP63, and ULBP2 (Figure 11).

### Differences in drug response between the low- and high-risk breast cancer groups

**Figure 12:**
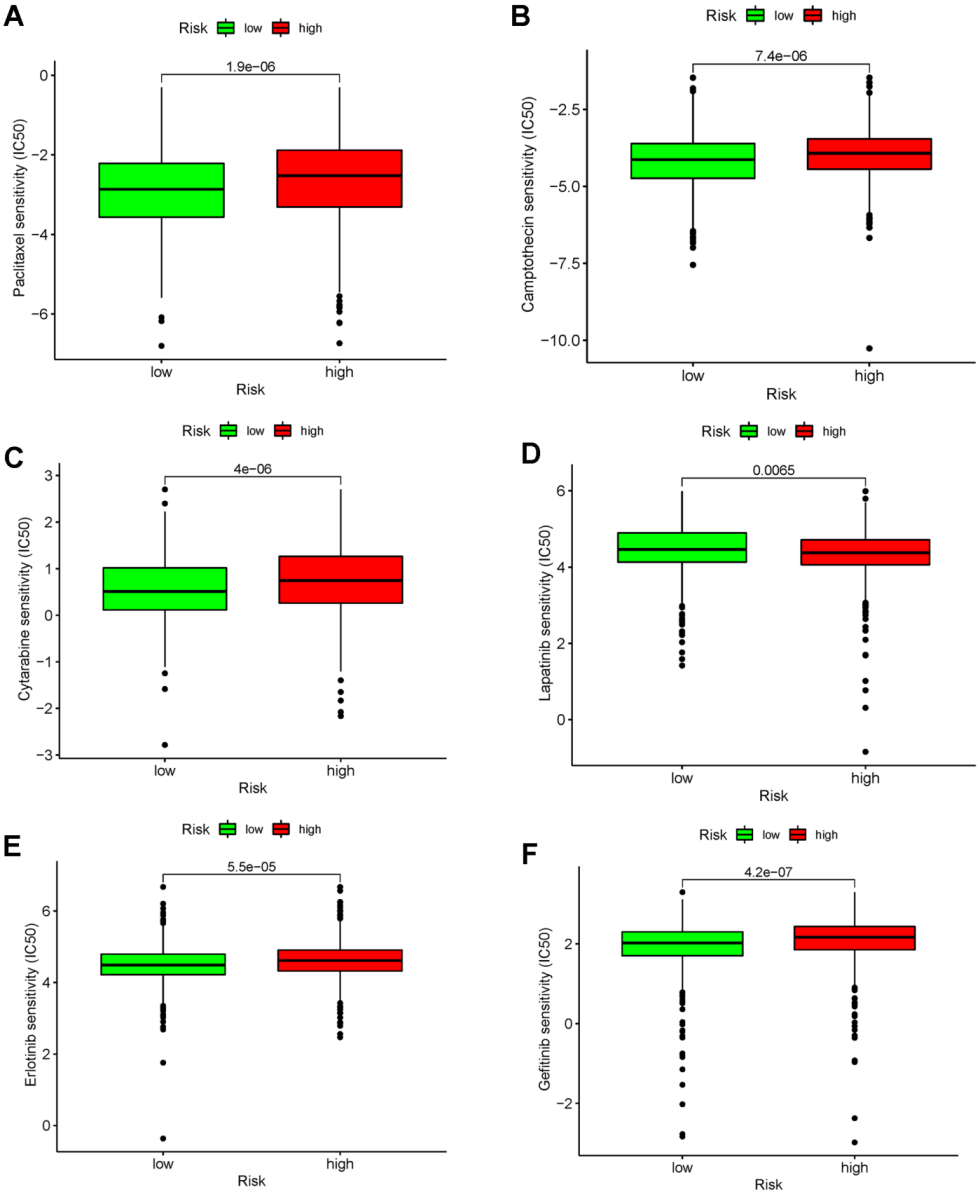
Durg-response analysis of (**A**) Paclitaxel. (**B**) Camptothecin. (**C**) Cytarabine. (**D**) Lapatinib. (**E**) Erlotinib. (**F**) Gefitinib.

As shown in Figure 12, the high-risk breast cancer cohort showed a significantly higher response to paclitaxel, cytarabine, camptothecin, erlotinib, and gefitinib, while the low-risk cohort showed a significantly better response to lapatinib.

## DISCUSSION

Breast cancer is a heterogeneous disease with a high incidence rate and poor prognosis [35]. As the molecular mechanism of breast cancer is complex, continuous studies aim to identify better molecular typing of breast cancer. Wang et al. [7] developed a five-gene (EDN2, CLEC3B, SV2C, WT1, and MUC2) prognostic signature using LASSO Cox regression analysis. Gao et al. [6] developed a pyroptosis-related lncRNA-associated (AC121761.2, AC027307.2, LINC01871, U73166.1, AL513477.2, AC005034.5 and AL451085.2) predictive model using LASSO Cox regression analysis. However, these risk models only considered the molecular status. Indeed, breast cancer is a complicated disease that requires additional considerations. Our nomogram is a comprehensive prognostic prediction tool that includes clinical characteristics, NMF clustering-based typing, and the risk score. Many multigene analysis-based models have been published in the last decade [36–38]. NMF clustering is a novel typing method that is rarely used in breast cancer and with which we can achieve a more detailed typing to predict more accurate prognoses for breast cancer patients.

Based on the shared DEG expression and survival data of breast cancer patients, eight novel subtypes were identified. According to the K-M survival plots, C4 and C5 breast cancer had a better OS and PFS, whereas C6 had an obviously poor prognosis. Notably, C4 and C5 breast cancer had high neutrophil infiltration, while C6 breast cancer had lower levels of neutrophil infiltration. As one of the most important immune cell types, neutrophils play a vital role in cancer progression, such as by directly eliminating cancer cells, releasing factors that affect the tumor microenvironment (TME), and producing reactive oxygen and nitrogen species [39]. These anti-tumor effects might account for the better prognosis of C4 and C5 breast cancer. Moreover, we found that C4 and C5 breast cancer had a low number of monocytes and that C6 breast cancer had highest monocyte infiltration. Mononuclear cells are precursors of tumor-associated macrophages (TAMs) which comprise the most abundant proportion of tumor-infiltrating immune cells [40]. Substantial evidence showed that TAMs are highly associated with poor prognosis in cancer [41, 42]. The potential mechanism of TAMs is complicated and includes tumor promotion, an increase in cancer resistance, and promotion of cancer cell migration [43–46]. Undoubtedly, mononuclear cells are important in the breast cancer microenvironment, as they have the potential to predict prognosis and become a therapeutic target.

To construct a stable predictive model, we then conducted LASSO Cox regression analysis with DEGs to divide the breast cancer cases into low- and high-risk subsets. Finally, 17 DEGs were included in the risk score calculation. Next, we reviewed previous studies of these 17 DEGs (Table 2). Although the functions of some of these 17 DEGs were unclear and even controversial, and not all of them were reported to be related to breast cancer, we plan to perform additional research on these DEGs. To explore the potential signaling pathways among these 17 DEGs in the low- and high-risk groups, we performed the GSEA, from which we found that high-risk breast cancer was associated with histidine metabolism, protein export, and steroid hormone biosynthesis. Matboli et al. [47] demonstrated that histidine-rich glycoprotein expression was higher in basal-like breast cancer than in the normal-like subtype, while other evidence demonstrated that the basal-like breast cancer subtype had a worse prognosis [48]. Furthermore, Saha et al. [49] reported that steroid hormone receptors can drive cell cycle regulation and breast cancer progression, thereby controlling tumor proliferation. These enriched pathways suggested that the disease included in the high-risk group was more invasive and was associated with poorer survival than that in the low-risk group.

**Table 2:** Differentially expressed genes in the risk score calculation formula.

**Gene**	**Protein name**	**Gene bank accession number**	**Function**
WNT7B [56, 57]	Wnt7b protein	AY400071	WNT7B is involved in tumor growth promotion, immunosuppression, angiogenesis, and cancer cell dissemination.
LEF1 [58]	Lymphoid enhancer binding factor 1	AY129650	LEF1 regulates glutathione metabolism, increases chemotherapy resistance, and promotes breast cancer brain metastasis.
BCL2A1 [59, 60]	BCL2 related protein A1	DQ081729	BCL2A1 represses hypoxia-induced cell death and mitochondria-mediated apoptosis and promotes tumor growth and metastasis.
ULBP2 [61, 62]	UL16 binding protein 2	AY358665	ULBP2 increases NK cell cytotoxicity resistance and promotes cervical cancer proliferation, invasion, and migration.
GABRQ [63, 64]	Gamma-aminobutyric acid type A receptor subunit theta	KJ899212	GABRQ promotes hepatocellular cancer cell proliferation.
FXYD3 [65]	FXYD domain containing ion transport regulator 3	KJ891826	FXYD3 promotes breast cancer cell proliferation.
SCG2 [66]	Secretogranin II	KJ897788	SCG2 enhances endothelial angiogenesis.
FOXJ1 [67–70]	Forkhead box J1	KJ891181	FOXJ1 promotes bladder cancer, prostate cancer, hepatocellular cancer, and gastric cancer growth, and metastasis.
TP63 [71]	Tumor protein p63	KR711025	TP63 increases expression of epidermal growth factor receptor in breast cancer and increases the response of breast cancer to cisplatin.
RYR1 [72]	Ryanodine receptor 1	AH006668	RYR1 plays a vital role as a calcium channel in excitation-contraction coupling in muscle.
FEZ1 [73–76]	Fasciculation and elongation protein zeta 1	AF123653	FEZ1 suppresses prostate, esophageal, gastric, bladder, and breast cancer progression, and mediates promoter methylation-mediated transcriptional downregulation and mitosis inhibition.
NRG1 [77]	Neuregulin 1	CR450288	Heregulin isoforms encoded by NRG1 promote tumor growth and induce metastasis.
NFE2 [78]	Nuclear factor, erythroid 2	CR450284	NFE2 promotes breast cancer cell growth in the bone microenvironment, which leads to bone metastasis; enhances expression of Wnt-related molecules.
HOXC13 [79]	Homeobox C13	AF263466	HOXC13 facilitates cervical cancer cell proliferation, migration, invasion and glycolysis through the β-catenin/c-Myc signaling pathway.
MMP25 [80]	Matrix metallopeptidase 25	HF584190	High expression of MMP25 in head and neck cancer is associated with a worse prognosis; MMP25 is related to apoptosis, the KRS signaling pathway, the PI3K/AKT/mTOR signaling pathway, and the JAK/STAT signaling pathway.
DTX1 [81, 82]	Deltex E3 ubiquitin ligase 1	KT584324	DTX1 is a regulator of the Notch signaling pathway and acts as an E3 ubiquitin ligase that can repress Notch gene expression and inhibit early-stage non-small cell lung carcinoma growth.

After further analysis, we found that the risk score was significantly correlated with clinical characteristics. Patients ≤60 years of age with stage I-II, M0, and N0-1 breast cancer had significantly lower risk score than those >60 years with stage III-IV, M1, and N2-3 breast cancer. Moreover, we found that T4 breast cancer had the highest risk score, followed by T2-3 and T1. Notably, no significant differences were identified between stages I and II, stages III and IV, T2 and T3, N0 and N1, or N2 and N3. We performed a correlation analysis for the risk score and several known breast cancer-associated genes, after which we found that TP53, KIT, MCL1, MAP3K1, JAK1, PDCD1, CTLA4, and CD274 were significantly negatively correlated with the risk score. The risk score was also negatively correlated with T cells, CD8+ T cells, cytotoxic lymphocytes, B lineage cells, NK cells, monocytic lineage cells, and myeloid dendritic cells, and was positively correlated with fibroblasts. From this analysis, we believed that the risk score was strongly associated with the TME. In breast cancer, many immune-related pathways are abnormally regulated, thereby influencing the microenvironment, such as through immune cell infiltration [50]. Based on current literature, some believe that the TME is a potential treatment target in breast cancer [51, 52]. To further demonstrate this conclusion, a drug sensitivity analysis was performed through which we identified significantly higher responses to agents, with the exception of lapatinib, in high-risk breast cancer. This special phenomenon was similar to what was observed in triple-negative breast cancer, which exhibited good chemotherapeutic sensitivity but poor prognosis [53–55]. Obviously, the risk score was strongly associated with the TME and was shown to predict chemotherapeutic and immunotherapeutic responses in breast cancer. However, no significant correlation was found between the risk score and breast cancer TMB, which requires additional research.

Based on these analyses, we believe that the risk score is valuable, but nevertheless, as a clinical predictive model, it should be better. Therefore, to further refine our model, we considered the risk score, clinical characteristics, and NMF clustering and constructed a nomogram. By summing the scores of these terms, we can predict the 1-, 3-, 5-year survival for every breast cancer patient, which might be a more precise and stable method. However, our analysis still has some limitations. First, our analysis only uses data from online databases, more real-word data are needed to further confirm our findings. Second, the detection of 17 DEGs would cost more than a model with fewer genes. However, our final prediction model has been confirmed to be effective, and since there are several other clinical prediction tools that require detection of more than 17 genes, our model is acceptable. Since breast cancer has varied subtypes, more detailed prediction models for the different subtypes should be constructed. We believe that these prediction models will be more powerful and cost-effective. Finally, although the HR of a single risk score was not very high, significant differences in OS and PFS as well as in AUC values were observed between the low- and high-risk score groups. To further improve the power of prediction model, we constructed a model using NMF clustering, the risk score, and clinical characteristics. Fortunately, our final nomogram model is shown to be better than any single factor.

In this study, we developed a novel predictive model using NMF clustering, clinical characteristics of breast cancer, and risk score based on 17 DEGs. The model was verified by randomly dividing the TCGA cohort into training and test cohorts, and separately analyzing the survival differences between low- and high-risk groups in these cohorts. Differences were observed in immune cell infiltration, clinical correlation, potential signaling pathways, and drug sensitivity.

## Supplementary Material

Supplementary File 1
