# Supplementary material for: Tumor microenvironment-related multigene prognostic prediction model for breast cancer
Source: Aging (Albany NY). 2022 Jan 20;14(2):845–68. doi: 10.18632/aging.203845 (PMC8833129; doi:10.18632/aging.203845)
Supplement: Supplementary File 1 [file aging-14-203845-s001.docx]

**Supplementary File 1.** **Codes for workflow.**

1. **Differential expressed genes analysis: limma (https://bioconductor.org/packages/release/bioc/html/limma.html) R package**

**Codes:**

group=sapply(strsplit(colnames(data),"\\-"), "[", 4)

group=sapply(strsplit(group,""), "[", 1)

group=gsub("2", "1", group)

conNum=length(group[group==1])

treatNum=length(group[group==0])

Type=c(rep(1,conNum), rep(2,treatNum))

outTab=data.frame()

for(i in row.names(data)){

rt=data.frame(expression=data[i,], Type=Type)

wilcoxTest=wilcox.test(expression ~ Type, data=rt)

pvalue=wilcoxTest$p.value

conGeneMeans=mean(data[i,1:conNum])

treatGeneMeans=mean(data[i,(conNum+1):ncol(data)])

logFC=log2(treatGeneMeans)-log2(conGeneMeans)

conMed=median(data[i,1:conNum])

treatMed=median(data[i,(conNum+1):ncol(data)])

diffMed=treatMed-conMed

if( ((logFC>0) & (diffMed>0)) | ((logFC<0) & (diffMed<0)) ){

outTab=rbind(outTab,cbind(gene=i,conMean=conGeneMeans,treatMean=treatGeneMeans,logFC=logFC,pValue=pvalue))

}

}

pValue=outTab[,"pValue"]

fdr=p.adjust(as.numeric(as.vector(pValue)), method="fdr")

outTab=cbind(outTab, fdr=fdr)

1. **Pheatmap (https://cran.r-project.org/web/packages/pheatmap/index.html) R package**

**Codes:**

outTab$fdr=as.numeric(outTab$fdr)

outTab$logFC=as.numeric(outTab$logFC)

Significant=ifelse((outTab$fdr<fdrFilter & abs(outTab$logFC)>logFCfilter), ifelse(outTab$logFC>logFCfilter,"Up","Down"), "Not")

p = ggplot(outTab, aes(logFC, -log10(fdr)))+

geom_point(aes(col=Significant))+

scale_color_manual(values=c("green", "black", "red"))+

labs(title = " ")+

theme(plot.title = element_text(size = 16, hjust = 0.5, face = "bold"))

p=p+theme_bw()

pdf("x.pdf", width=6.2, height=5.5)

print(p)

dev.off()

1. **Ggplot2 (https://cran.r-project.org/web/packages/ggplot2/index.html) R package**

**Codes:**

hmExp=log2(data[as.vector(outDiff[,1]),]+0.01)

Type=c(rep("N",conNum),rep("T",treatNum))

names(Type)=colnames(data)

Type=as.data.frame(Type)

pdf(file="x.pdf",width=10,height=8)

pheatmap(hmExp,

annotation=Type,

color = colorRampPalette(c(rep("blue",3), "white", rep("red",3)))(50),

cluster_cols =F,

scale="row",

show_colnames = F,

show_rownames = T,

fontsize = 8,

fontsize_row=3,

fontsize_col=8)

dev.off()

1. **Protein-protein interaction network (https://string-db.org/)**
2. **Hub genes barplot**

**Codes:**

n=as.matrix(tb)[1:30,]

pdf(file="x.pdf",width=8,height=6)

par(mar=c(3,10,3,3),xpd=T)

bar=barplot(n,horiz=TRUE,col="skyblue",names=FALSE)

text(x=n-0.5,y=bar,n)

text(x=-0.2,y=bar,label=names(n),xpd=T,pos=2)

dev.off()

1. **GO enrichment analysis: clusterprofiler (http://bioconductor.org/packages/release/bioc/html/clusterProfiler.html) R package**

**Codes:**

kk=enrichGO(gene=gene, OrgDb=org.Hs.eg.db, pvalueCutoff=1, qvalueCutoff=1, ont="all", readable=T)

GO=as.data.frame(kk)

GO=GO[(GO$pvalue<pvalueFilter & GO$qvalue<qvalueFilter),]

write.table(GO, file="GO.txt", sep="\t", quote=F, row.names = F)

showNum=10

if(nrow(GO)<30){

showNum=nrow(GO)

}

pdf(file="y.pdf", width=10, height=7)

bar=barplot(kk, drop=TRUE, showCategory=showNum, split="ONTOLOGY", color=colorSel) + facet_grid(ONTOLOGY~., scale='free')

print(bar)

dev.off()

pdf(file="x.pdf", width=10, height=7)

bub=dotplot(kk, showCategory=showNum, orderBy="GeneRatio", split="ONTOLOGY", color=colorSel) + facet_grid(ONTOLOGY~., scale='free')

print(bub)

dev.off()

1. **KEGG enrichment analysis: clusterprofiler (http://bioconductor.org/packages/release/bioc/html/clusterProfiler.html) R package**

**Codes:**

kk=enrichKEGG(gene=gene, organism="hsa", pvalueCutoff=1, qvalueCutoff=1)

KEGG=as.data.frame(kk)

KEGG$geneID=as.character(sapply(KEGG$geneID,function(x)paste(rt$genes[match(strsplit(x,"/")[[1]],as.character(rt$entrezID))],collapse="/")))

KEGG=KEGG[(KEGG$pvalue<pvalueFilter & KEGG$qvalue<qvalueFilter),]

write.table(KEGG, file="KEGG.txt", sep="\t", quote=F, row.names = F)

showNum=30

if(nrow(KEGG)<showNum){

showNum=nrow(KEGG)

}

pdf(file="y.pdf", width=9, height=7)

barplot(kk, drop=TRUE, showCategory=showNum, color=colorSel)

dev.off()

pdf(file="x.pdf", width = 9, height = 7)

dotplot(kk, showCategory=showNum, orderBy="GeneRatio", color=colorSel)

dev.off()

1. **NMF (https://cran.r-project.org/web/packages/NMF/index.html) R package**

**survival (https://cran.r-project.org/web/packages/survival/index.html) R package**

**Codes:**

sigGenes=c()

for(i in colnames(rt)[3:ncol(rt)]){

cox=coxph(Surv(futime, fustat) ~ rt[,i], data = rt)

coxSummary=summary(cox)

coxP=coxSummary$coefficients[,"Pr(>|z|)"]

if(coxP<0.01){ sigGenes=c(sigGenes,i) }

}

data=t(rt[,sigGenes])

res=nmf(data, rank=2:10, method="brunet", nrun=10, seed=123456)

pdf(file="x.pdf", width=8, height=7, onefile=F)

plot(res)

dev.off()

pdf(file="y.pdf", width=15, height=15, onefile=F)

consensusmap(res,

annRow=NA,

annCol=NA,

#tracks=c("consensus:"),

main="Consensus matrix",

info=FALSE)

dev.off()

clusterNum=8

res=nmf(data, rank=clusterNum, method="brunet", nrun=10, seed=123456)

Cluster=predict(res)

Cluster=as.data.frame(Cluster)

Cluster$Cluster=paste0("C", Cluster$Cluster)

clusterOut=rbind(ID=colnames(Cluster), Cluster)

write.table(clusterOut, file="cluster.txt", sep="\t", quote=F, col.names=F)

pdf(file="z.pdf", width=6, height=6, onefile=F)

consensusmap(res,

annRow=NA,

annCol=NA,

#tracks=c("consensus:"),

main="Consensus matrix",

info=FALSE)

dev.off()

1. **NMF clustering survival analysis**

**Codes:** bioCol=c("#0066FF","#FF0000","#FF9900","#6E568C","#7CC767","#223D6C","#D20A13","#FFD121","#088247","#11AA4D")

bioCol=bioCol[1:length]

surPlot=ggsurvplot(fit,

data=rt,

conf.int=F,

pval=pValue,

pval.size=6,

legend.title="Cluster",

legend.labs=levels(factor(rt[,"Cluster"])),

legend = c(0.8, 0.8),

font.legend=10,

xlab="Time(years)",

ylab="Overall survival",

break.time.by = 2,

palette = bioCol,

#surv.median.line = "hv",

risk.table=T,

cumevents=F,

risk.table.height=.3)

pdf(file="x.pdf",onefile = FALSE,width=7,height=6)

print(surPlot)

dev.off()

1. **NMF clustering immune infiltration analysis: ggpubr (https://cran.r-project.org/web/packages/ggpubr/index.html) R package**

**Codes:**

bioCol=c("#0066FF","#FF0000","#FF9900","#6E568C","#7CC767","#223D6C","#D20A13","#FFD121","#088247","#11AA4D")

bioCol=bioCol[1:8]

for(i in colnames(data)[1:(ncol(data)-1)]){

data[,i][data[,i]>quantile(data[,i],0.99)]=quantile(data[,i],0.99)

violin=ggviolin(data, x="Cluster", y=i, fill = "Cluster",

xlab="", ylab=i,

legend.title="Cluster",

palette=bioCol,

pval=pValue,

pval.size=6,

add = "boxplot", add.params = list(fill="white"))+

stat_compare_means(comparisons = my_comparisons)

#stat_compare_means(comparisons = my_comparisons,symnum.args=list(cutpoints = c(0, 0.001, 0.01, 0.05, 1), symbols = c("***", "**", "*", "ns")),label = "p.signif")

pdf(file=paste0("violin.", i, ".pdf"), width=5, height=4.5)

print(violin)

dev.off()

}

1. **LASSO regression analysis: survival (https://cran.r-project.org/web/packages/survival/index.html), caret (https://topepo.github.io/caret/), glmnet (https://cran.r-project.org/web/packages/glmnet/index.html), survminer (https://cran.r-project.org/web/packages/survminer/index.html), timeROC (https://cran.r-project.org/web/packages/timeROC/index.html) R packages**

**Codes:**

inTrain=createDataPartition(y=rt[,3], p=0.7, list=F)

train=rt[inTrain,]

test=rt[-inTrain,]

trainOut=cbind(id=row.names(train),train)

testOut=cbind(id=row.names(test),test)

outUniTab=data.frame()

sigGenes=c("futime","fustat")

for(i in colnames(train[,3:ncol(train)])){

cox <- coxph(Surv(futime, fustat) ~ train[,i], data = train)

coxSummary = summary(cox)

coxP=coxSummary$coefficients[,"Pr(>|z|)"]

if(coxP<coxPfilter){

sigGenes=c(sigGenes,i)

outUniTab=rbind(outUniTab,

cbind(id=i,

HR=coxSummary$conf.int[,"exp(coef)"],

HR.95L=coxSummary$conf.int[,"lower .95"],

HR.95H=coxSummary$conf.int[,"upper .95"],

pvalue=coxSummary$coefficients[,"Pr(>|z|)"])

)

}

}

uniSigExp=train[,sigGenes]

uniSigExpOut=cbind(id=row.names(uniSigExp),uniSigExp)

if(length(sigGenes)<5){next}

x=as.matrix(uniSigExp[,c(3:ncol(uniSigExp))])

y=data.matrix(Surv(uniSigExp$futime,uniSigExp$fustat))

fit <- glmnet(x, y, family = "cox", maxit = 1000)

cvfit <- cv.glmnet(x, y, family="cox", maxit = 1000)

coef <- coef(fit, s = cvfit$lambda.min)

index <- which(coef != 0)

actCoef <- coef[index]

lassoGene=row.names(coef)[index]

lassoSigExp=uniSigExp[,c("futime", "fustat", lassoGene)]

lassoSigExpOut=cbind(id=row.names(lassoSigExp), lassoSigExp)

geneCoef=cbind(Gene=lassoGene, Coef=actCoef)

if(nrow(geneCoef)<2){next}

multiCox <- coxph(Surv(futime, fustat) ~ ., data = lassoSigExp)

multiCox=step(multiCox,direction = "both")

multiCoxSum=summary(multiCox)

outMultiTab=data.frame()

outMultiTab=cbind(

coef=multiCoxSum$coefficients[,"coef"],

HR=multiCoxSum$conf.int[,"exp(coef)"],

HR.95L=multiCoxSum$conf.int[,"lower .95"],

HR.95H=multiCoxSum$conf.int[,"upper .95"],

pvalue=multiCoxSum$coefficients[,"Pr(>|z|)"])

outMultiTab=cbind(id=row.names(outMultiTab),outMultiTab)

riskScore=predict(multiCox,type="risk",newdata=train)

coxGene=rownames(multiCoxSum$coefficients)

coxGene=gsub("`","",coxGene)

outCol=c("futime","fustat",coxGene)

medianTrainRisk=median(riskScore)

risk=as.vector(ifelse(riskScore>medianTrainRisk,"high","low"))

trainRiskOut=cbind(id=rownames(cbind(train[,outCol],riskScore,risk)),cbind(train[,outCol],riskScore,Risk=risk))

riskScoreTest=predict(multiCox,type="risk",newdata=test)

riskTest=as.vector(ifelse(riskScoreTest>medianTrainRisk,"high","low"))

testRiskOut=cbind(id=rownames(cbind(test[,outCol],riskScoreTest,riskTest)),cbind(test[,outCol],riskScore=riskScoreTest,Risk=riskTest))

diff=survdiff(Surv(futime, fustat) ~Risk,data = trainRiskOut)

pValue=1-pchisq(diff$chisq, df=1)

diffTest=survdiff(Surv(futime, fustat) ~Risk,data = testRiskOut)

pValueTest=1-pchisq(diffTest$chisq, df=1)

predictTime=1

roc=timeROC(T=train$futime, delta=train$fustat,

marker=riskScore, cause=1,

times=c(predictTime), ROC=TRUE)

rocTest=timeROC(T=test$futime, delta=test$fustat,

marker=riskScoreTest, cause=1,

times=c(predictTime), ROC=TRUE)

write.table(trainOut,file="data.train.txt",sep="\t",quote=F,row.names=F)

write.table(testOut,file="data.test.txt",sep="\t",quote=F,row.names=F)

write.table(outUniTab,file="uni.trainCox.txt",sep="\t",row.names=F,quote=F)

write.table(uniSigExpOut,file="uni.SigExp.txt",sep="\t",row.names=F,quote=F)

write.table(lassoSigExpOut,file="lasso.SigExp.txt",sep="\t",row.names=F,quote=F)

pdf("lasso.lambda.pdf")

plot(fit, xvar = "lambda", label = TRUE)

dev.off()

pdf("lasso.cvfit.pdf")

plot(cvfit)

abline(v=log(c(cvfit$lambda.min,cvfit$lambda.1se)), lty="dashed")

dev.off()

outMultiTab=outMultiTab[,1:2]

write.table(outMultiTab,file="multiCox.txt",sep="\t",row.names=F,quote=F)

write.table(trainRiskOut,file="risk.TCGAtrain.txt",sep="\t",quote=F,row.names=F)

write.table(testRiskOut,file="risk.TCGAtest.txt",sep="\t",quote=F,row.names=F)

allRiskOut=rbind(trainRiskOut, testRiskOut)

write.table(allRiskOut,file="risk.TCGAall.txt",sep="\t",quote=F,row.names=F)

1. **Low- and high-risk score groups survival analysis: survival (https://cran.r-project.org/web/packages/survival/index.html), survminer (https://cran.r-project.org/web/packages/survminer/index.html) R package**

**Codes**:

surPlot=ggsurvplot(fit,

data=rt,

conf.int=T,

pval=pValue,

pval.size=6,

legend.title="Risk",

legend.labs=c("High risk", "Low risk"),

xlab="Time(years)",

break.time.by = 1,

palette=c("red", "blue"),

risk.table=TRUE,

risk.table.title="",

risk.table.col = "strata",

risk.table.height=.25)

pdf(file=outFile,onefile = FALSE,width = 6.5,height =5.5)

print(surPlot)

dev.off()

}

bioSurvival(inputFile="risk.TCGAtrain.txt", outFile="surv.TCGAtrain.pdf")

bioSurvival(inputFile="risk.TCGAtest.txt", outFile="surv.TCGAtest.pdf")

bioSurvival(inputFile="risk.TCGAall.txt", outFile="surv.TCGAall.pdf")

1. **Receiver operator characteristic curve (ROC):** **survival (https://cran.r-project.org/web/packages/survival/index.html), survminer (https://cran.r-project.org/web/packages/survminer/index.html), timeROC (https://cran.r-project.org/web/packages/timeROC/index.html) R packages**

**Codes**:

ROC_rt=timeROC(T=rt$futime,delta=rt$fustat,

marker=rt$riskScore,cause=1,

weighting='aalen',

times=c(1,3,5),ROC=TRUE)

pdf(file=rocFile,width=5,height=5)

plot(ROC_rt,time=1,col='green',title=FALSE,lwd=2)

plot(ROC_rt,time=3,col='blue',add=TRUE,title=FALSE,lwd=2)

plot(ROC_rt,time=5,col='red',add=TRUE,title=FALSE,lwd=2)

legend('bottomright',

c(paste0('AUC at 1 years: ',sprintf("%.03f",ROC_rt$AUC[1])),

paste0('AUC at 3 years: ',sprintf("%.03f",ROC_rt$AUC[2])),

paste0('AUC at 5 years: ',sprintf("%.03f",ROC_rt$AUC[3]))),

col=c("green",'blue','red'),lwd=2,bty = 'n')

dev.off()

}

bioROC(inputFile="risk.TCGAtrain.txt", rocFile="ROC.TCGAtrain.pdf")

bioROC(inputFile="risk.TCGAtest.txt", rocFile="ROC.TCGAtest.pdf")

bioROC(inputFile="risk.TCGAall.txt", rocFile="ROC.TCGAall.pdf")

1. **Clinical characteristics correlation analysis: survival (https://cran.r-project.org/web/packages/survival/index.html), survminer (https://cran.r-project.org/web/packages/survminer/index.html) R packages**

**Codes:**

for(j in names(tab)){

rt1=rt[(rt[,"clinical"]==j),]

tab1=table(rt1[,"Risk"])

tab1=tab1[tab1!=0]

labels=names(tab1)

if(length(labels)!=2){next}

if((cliName=="x") | (cliName=="x") | (cliName=="X")){

titleName=paste0("x",j)

}

diff=survdiff(Surv(futime, fustat) ~Risk,data = rt1)

pValue=1-pchisq(diff$chisq,df=1)

if(pValue<0.001){

pValue="p<0.001"

}else{

pValue=paste0("p=",sprintf("%.03f",pValue))

}

fit <- survfit(Surv(futime, fustat) ~ Risk, data = rt1)

surPlot=ggsurvplot(fit,

data=rt1,

conf.int=F,

pval=pValue,

pval.size=6,

title=paste0("Patients with ",j),

legend.title="Risk",

legend.labs=labels,

font.legend=12,

xlab="Time(years)",

break.time.by = 2,

palette=c("red", "blue"),

risk.table=F,

risk.table.title="",

risk.table.col = "strata",

risk.table.height=.25)

j=gsub(">=","ge",j);j=gsub("<=","le",j);j=gsub(">","gt",j);j=gsub("<","lt",j)

pdf(file=paste0("survival.",cliName,"_",j,".pdf"), onefile = FALSE,

width = 6,

height =5)

print(surPlot)

dev.off()

}

1. **Univariate Cox analysis and multivariate Cox analysis**

**Codes:**

uniTab=data.frame()

for(i in colnames(rt[,3:ncol(rt)])){

cox <- coxph(Surv(futime, fustat) ~ rt[,i], data = rt)

coxSummary = summary(cox)

uniTab=rbind(uniTab,

cbind(id=i,

HR=coxSummary$conf.int[,"exp(coef)"],

HR.95L=coxSummary$conf.int[,"lower .95"],

HR.95H=coxSummary$conf.int[,"upper .95"],

pvalue=coxSummary$coefficients[,"Pr(>|z|)"])

)

}

write.table(uniTab,file=uniOutFile,sep="\t",row.names=F,quote=F)

uniTab=uniTab[as.numeric(uniTab[,"pvalue"])<0.05,]

rt1=rt[,c("futime","fustat",as.vector(uniTab[,"id"]))]

multiCox=coxph(Surv(futime, fustat) ~ ., data = rt1)

multiCoxSum=summary(multiCox)

multiTab=data.frame()

multiTab=cbind(

HR=multiCoxSum$conf.int[,"exp(coef)"],

HR.95L=multiCoxSum$conf.int[,"lower .95"],

HR.95H=multiCoxSum$conf.int[,"upper .95"],

pvalue=multiCoxSum$coefficients[,"Pr(>|z|)"])

multiTab=cbind(id=row.names(multiTab),multiTab)

write.table(multiTab,file=multiOutFile,sep="\t",row.names=F,quote=F)

}

1. **Nomogram model construction: survival (https://cran.r-project.org/web/packages/survival/index.html), regplot (https://cran.r-project.org/web/packages/regplot/index.html), rms (https://cran.r-project.org/web/packages/rms/index.html)**

**Codes:**

res.cox=coxph(Surv(futime, fustat) ~ . , data = rt)

nom1=regplot(res.cox,

plots = c("density", "boxes"),

clickable=F,

title="",

points=TRUE,

droplines=TRUE,

observation=rt[1,],

rank="sd",

failtime = c(1,3,5),

prfail = F)

nomoRisk=predict(res.cox, data=rt, type="risk")

rt=cbind(risk1, Nomogram=nomoRisk)

outTab=rbind(ID=colnames(rt), rt)

write.table(outTab, file="nomoRisk.txt", sep="\t", col.names=F, quote=F)

pdf(file="calibration.pdf", width=5, height=5)

f <- cph(Surv(futime, fustat) ~ Nomogram, x=T, y=T, surv=T, data=rt, time.inc=1)

cal <- calibrate(f, cmethod="KM", method="boot", u=1, m=(nrow(rt)/3), B=1000)

plot(cal, xlim=c(0,1), ylim=c(0,1),

xlab="Nomogram-predicted OS (%)", ylab="Observed OS (%)", lwd=1.5, col="green", sub=F)

f <- cph(Surv(futime, fustat) ~ Nomogram, x=T, y=T, surv=T, data=rt, time.inc=3)

cal <- calibrate(f, cmethod="KM", method="boot", u=3, m=(nrow(rt)/3), B=1000)

plot(cal, xlim=c(0,1), ylim=c(0,1), xlab="", ylab="", lwd=1.5, col="blue", sub=F, add=T)

f <- cph(Surv(futime, fustat) ~ Nomogram, x=T, y=T, surv=T, data=rt, time.inc=5)

cal <- calibrate(f, cmethod="KM", method="boot", u=5, m=(nrow(rt)/3), B=1000)

plot(cal, xlim=c(0,1), ylim=c(0,1), xlab="", ylab="", lwd=1.5, col="red", sub=F, add=T)

legend('bottomright', c('1-year', '3-year', '5-year'),

col=c("green","blue","red"), lwd=1.5, bty = 'n')

dev.off()

1. **Decision curve analysis: survival (https://cran.r-project.org/web/packages/survival/index.html), survminer (https://cran.r-project.org/web/packages/survminer/index.html), timeROC (https://cran.r-project.org/web/packages/timeROC/index.html), ggDCA (https://cran.r-project.org/web/packages/ggDCA/index.html)**

**Codes:**

rt=cbind(risk1[,c("futime","fustat","Risk","Nomogram")], cli1)

rt[,"Age"]=ifelse(rt[,"Age"]>60, 1, 0)

rt[,"Nomogram"]=ifelse(rt[,"Nomogram"]>median(rt[,"Nomogram"]), 1, 0)

Nomogram<-coxph(Surv(futime,fustat)~Nomogram,rt)

Risk<-coxph(Surv(futime,fustat)~Risk,rt)

Age<-coxph(Surv(futime,fustat)~Age,rt)

Stage<-coxph(Surv(futime,fustat)~Stage,rt)

T<-coxph(Surv(futime,fustat)~T,rt)

M<-coxph(Surv(futime,fustat)~M,rt)

N<-coxph(Surv(futime,fustat)~N,rt)

pdf(file="DCA.pdf", width=6.5, height=5.2)

d_train=dca(Nomogram,Risk,Age,Stage,T,M,N, times=predictTime,xstat=0.00,xstop=0.03,xby=0.01)

ggplot(d_train, linetype=1)

dev.off()

rt=cbind(risk1[,c("futime","fustat","riskScore","Nomogram")], cli1)

aucText=c()

bioCol=rainbow(ncol(rt)-1, s=0.9, v=0.9)

pdf(file="cliROC.pdf", width=6, height=6)

i=3

ROC_rt=timeROC(T=risk$futime,

delta=risk$fustat,

marker=risk$riskScore, cause=1,

weighting='aalen',

times=c(predictTime),ROC=TRUE)

plot(ROC_rt, time=predictTime, col=bioCol[i-2], title=FALSE, lwd=2)

aucText=c(paste0("Risk", ", AUC=", sprintf("%.3f",ROC_rt$AUC[2])))

abline(0,1)

for(i in 5:ncol(rt)){

ROC_rt=timeROC(T=rt$futime,

delta=rt$fustat,

marker=rt[,i], cause=1,

weighting='aalen',

times=c(predictTime),ROC=TRUE)

plot(ROC_rt, time=predictTime, col=bioCol[i-2], title=FALSE, lwd=2, add=TRUE)

aucText=c(aucText, paste0(colnames(rt)[i],", AUC=",sprintf("%.3f",ROC_rt$AUC[2])))

}

legend("bottomright", aucText,lwd=2,bty="n",col=bioCol[1:(ncol(rt)-1)])

dev.off()

1. **Gene set enrichment analysis: limma (https://bioconductor.org/packages/release/bioc/html/limma.html), org.Hs.eg.db (https://bioconductor.org/packages/release/data/annotation/html/org.Hs.eg.db.html), clusterprofiler (http://bioconductor.org/packages/release/bioc/html/clusterProfiler.html), enrichplot (https://bioconductor.org/packages/release/bioc/html/enrichplot.html)**

**Codes:**

kk=GSEA(logFC, TERM2GENE=gmt, pvalueCutoff = 1)

kkTab=as.data.frame(kk)

kkTab=kkTab[kkTab$pvalue<0.05,]

write.table(kkTab,file="GSEA.result.txt",sep="\t",quote=F,row.names = F)

termNum=5

kkUp=kkTab[kkTab$NES>0,]

if(nrow(kkUp)>=termNum){

showTerm=row.names(kkUp)[1:termNum]

gseaplot=gseaplot2(kk, showTerm, base_size=8, title="Enriched in high risk group")

pdf(file="GSEA.highRisk.pdf", width=7, height=5.5)

print(gseaplot)

dev.off()

}

termNum=5

kkDown=kkTab[kkTab$NES<0,]

if(nrow(kkDown)>=termNum){

showTerm=row.names(kkDown)[1:termNum]

gseaplot=gseaplot2(kk, showTerm, base_size=8, title="Enriched in low risk group")

pdf(file="GSEA.lowRisk.pdf", width=7, height=5.5)

print(gseaplot)

dev.off()

}

1. **Correction analysis: limma (https://bioconductor.org/packages/release/bioc/html/limma.html), ggpubr (https://cran.r-project.org/web/packages/ggpubr/index.html), corrplot (https://cran.r-project.org/web/packages/corrplot/index.html), circlize (https://cran.r-project.org/web/packages/circlize/index.html), ggalluvial (https://cran.r-project.org/web/packages/ggalluvial/index.html), ggplot2 (https://cran.r-project.org/web/packages/ggplot2/index.html), dplyr (https://cran.r-project.org/web/packages/dplyr/index.html) R packages**

**Codes:**

for(clinical in colnames(rt[,2:ncol(rt)])){

data=rt[c("riskScore", clinical)]

colnames(data)=c("riskScore", "clinical")

data=data[(data[,"clinical"]!="unknow"),]

group=levels(factor(data$clinical))

data$clinical=factor(data$clinical, levels=group)

comp=combn(group,2)

my_comparisons=list()

for(i in 1:ncol(comp)){my_comparisons[[i]]<-comp[,i]}

boxplot=ggboxplot(data, x="clinical", y="riskScore", color="clinical",

xlab=clinical,

ylab="Risk score",

legend.title=clinical,

add = "jitter")+

stat_compare_means(comparisons = my_comparisons)

#stat_compare_means(comparisons = my_comparisons,symnum.args=list(cutpoints = c(0, 0.001, 0.01, 0.05, 1), symbols = c("***", "**", "*", "ns")),label = "p.signif")

pdf(file=paste0("cliCor.", clinical, ".pdf"), width=5.5, height=5)

print(boxplot)

dev.off()

}

rt=read.table(expFile, header=T, sep="\t", check.names=F)

rt=as.matrix(rt)

rownames(rt)=rt[,1]

exp=rt[,2:ncol(rt)]

dimnames=list(rownames(exp), colnames(exp))

data=matrix(as.numeric(as.matrix(exp)), nrow=nrow(exp), dimnames=dimnames)

data=avereps(data)

group=sapply(strsplit(colnames(data),"\\-"), "[", 4)

group=sapply(strsplit(group,""), "[", 1)

group=gsub("2", "1", group)

data=data[,group==0]

geneRT=read.table(geneFile, header=F, sep="\t", check.names=F)

data=data[as.vector(geneRT[,1]),]

data=t(data)

rownames(data)=gsub("(.*?)\\-(.*?)\\-(.*?)\\-.*", "\\1\\-\\2\\-\\3", rownames(data))

data=avereps(data)

data=log2(data+1)

risk=read.table(riskFile, header=T, sep="\t", check.names=F, row.names=1)

sameSample=intersect(row.names(data), row.names(risk))

data=cbind(risk[sameSample,"riskScore",drop=F], data[sameSample,,drop=F])

M=cor(data)

res1=cor.mtest(data, conf.level=0.95)

pdf(file="geneCor.pdf", width=7, height=7)

corrplot(M,

order="original",

method = "circle",

type = "upper",

tl.cex=1, pch=T,

p.mat = res1$p,

insig = "label_sig",

pch.cex = 1.6,

sig.level=0.05,

number.cex = 1,

col=colorRampPalette(c("blue", "white", "red"))(50),

tl.col="black")

dev.off()

pdf(file="immuneCor.pdf", width=7, height=7)

corrplot(M,

order="original",

method = "circle",

type = "upper",

tl.cex=0.8, pch=T,

p.mat = res1$p,

insig = "label_sig",

pch.cex = 1.6,

sig.level=0.05,

number.cex = 1,

col=colorRampPalette(c("blue", "white", "red"))(50),

tl.col="black")

dev.off()

col = c(rgb(1,0,0,seq(1,0,length=32)),rgb(0,1,0,seq(0,1,length=32)))

cor1[cor1==1]=0

c1 = ifelse(c(cor1)>=0,rgb(1,0,0,abs(cor1)),rgb(0,1,0,abs(cor1)))

col1 = matrix(c1,nc=ncol(rt))

pdf(file="circos.pdf", width=7, height=7)

par(mar=c(2,2,2,4))

circos.par(gap.degree=c(3,rep(2, nrow(cor1)-1)), start.degree = 180)

chordDiagram(cor1, grid.col=rainbow(ncol(rt)), col=col1, transparency = 0.5, symmetric = T)

par(xpd=T)

colorlegend(col, vertical = T,labels=c(1,0,-1),xlim=c(1.1,1.3),ylim=c(-0.4,0.4))

dev.off()

circos.clear()

pdf(file="ggalluvial.pdf", width=6, height=6)

mycol=rep(c("#029149","#6E568C","#E0367A","#D8D155","#223D6C","#D20A13","#431A3D","#91612D","#FFD121","#088247","#11AA4D","#58CDD9","#7A142C","#5D90BA","#64495D","#7CC767"),15)

ggplot(corLodes, aes(x = x, stratum = stratum, alluvium = Cohort,fill = stratum, label = stratum)) +

scale_x_discrete(expand = c(0, 0)) +

geom_flow(width = 2/10,aes.flow = "forward") +

geom_stratum(alpha = .9,width = 2/10) +

scale_fill_manual(values = mycol) +

geom_text(stat = "stratum", size = 3,color="black") +

xlab("") + ylab("") + theme_bw() +

theme(axis.line = element_blank(),axis.ticks = element_blank(),axis.text.y = element_blank()) +

theme(panel.grid =element_blank()) +

theme(panel.border = element_blank()) +

ggtitle("") + guides(fill = FALSE)

dev.off()

1. **Drug response analysis: limma (https://bioconductor.org/packages/release/bioc/html/limma.html), ggpubr (https://cran.r-project.org/web/packages/ggpubr/index.html), pRRophetic (https://github.com/paulgeeleher/pRRophetic), ggplot2 (https://cran.r-project.org/web/packages/ggplot2/index.html) R packages**

**Codes:**

senstivity=pRRopheticPredict(data, drug, selection=1)

senstivity=senstivity[senstivity!="NaN"]

senstivity[senstivity>quantile(senstivity,0.99)]=quantile(senstivity,0.99)

risk=read.table(riskFile,header=T, sep="\t",check.names=F, row.names=1)

sameSample=intersect(row.names(risk), names(senstivity))

risk=risk[sameSample,"risk",drop=F]

senstivity=senstivity[sameSample]

rt=cbind(risk,senstivity)

rt$risk=factor(rt$risk, levels=c("low", "high"))

type=levels(factor(rt[,"risk"]))

comp=combn(type, 2)

my_comparisons=list()

for(i in 1:ncol(comp)){my_comparisons[[i]]<-comp[,i]}

boxplot=ggboxplot(rt, x="risk", y="senstivity",fill="risk",

xlab="Risk",

ylab=paste0(drug, " senstivity (IC50)"),

legend.title="Risk",

palette=c("green", "red")

)+

stat_compare_means(comparisons=my_comparisons)

pdf(file=paste0(drug, ".pdf"),width=5, height=4.5)

print(boxplot)

dev.off()
